# An investigation into in‐sample and out‐of‐sample model selection for nonstationary autoregressive models

**DOI:** 10.1111/bmsp.70012

**Published:** 2025-10-28

**Authors:** Yong Zhang, Anja F. Ernst, Ginette Lafit, Ward B. Eiling, Laura F. Bringmann

**Affiliations:** ^1^ Department of Psychometrics and Statistics University of Groningen Groningen the Netherlands; ^2^ Methodology of Educational Sciences Research Group Faculty of Psychology and Educational Sciences, KU Leuven Leuven Belgium; ^3^ Department of Methodology and Statistics Faculty of Social and Behavioural Sciences, Utrecht University Utrecht the Netherlands

**Keywords:** cross‐validation, idiographic analysis, information criteria, model selection, nonstationarity, predictive accuracy, time‐series

## Abstract

The stationary autoregressive model forms an important base of time‐series analysis in today's psychology research. Diverse nonstationary extensions of this model are developed to capture different types of changing temporal dynamics. However, researchers do not always have a solid theoretical base to rely on for deciding a‐priori which of these nonstationary models is the most appropriate for a given time‐series. In this case, correct model selection becomes a crucial step to ensure an accurate understanding of the temporal dynamics. This study consists of two main parts. First, with a simulation study, we investigated the performance of in‐sample (information criteria) and out‐of‐sample (cross‐validation, out‐of‐sample prediction) model selection techniques in identifying six different univariate nonstationary processes. We found that the Bayesian information criteria (BIC) has an overall optimal performance whereas other techniques' performance depends largely on the time‐series' length. Then, we re‐analysed a 239‐day‐long time‐series of positive and negative affect to illustrate the model selection process. Combining the simulation results and practical considerations from the empirical analysis, we argue that model selection for nonstationary time‐series should not completely rely on data‐driven approaches. Instead, more theory‐driven approaches where researchers actively integrate their qualitative understanding will inform the data‐driven approaches.

## 1 INTRODUCTION

Psychological researchers increasingly use time‐series analysis to understand within‐person processes, for example, emotion dynamics (Hamaker et al., 2015; Kuppens & Verduyn, 2017; Reitsema et al., 2022) and the symptom dynamics of mental disorders (Borsboom & Cramer, 2013; Bringmann, 2021; Cramer et al., 2016; Fried & Cramer, 2017). Researchers often treat such within‐person dynamics as covariance‐stationary processes (Hamilton, 1994), meaning that they assume that the mean, variance and autocorrelation are stable over time (for simplicity, we will further refer to “covariance‐stationary” as “stationary” in the text; Koval et al., 2013). However, many clinical researchers question the assumption of stationarity and explicitly study the presence of changes in one's temporal dynamics. More specifically, they consider that changes in one's temporal dynamics are related to the improvement or worsening of the individual's mental health (Helmich et al., 2021, 2023; Schat et al., 2024; Schreuder et al., 2020, 2024; Wichers et al., 2016). For instance, effective depression treatment is expected to reduce the mean and autocorrelation of depressive symptoms (Koval et al., 2012, 2013). Following this approach, researchers indeed found that stationarity is not always a realistic assumption when studying such within‐person dynamics (Bringmann et al., 2017; Cabrieto et al., 2018; van de Leemput et al., 2014). Thus, assessing the potential nonstationarity in time‐series analysis is crucial for acquiring a more accurate understanding of the underlying dynamics.

In a systematic review of psychological studies that conducted idiographic analyses of intensive longitudinal data,^1^ we discovered that 50% of reviewed studies (60 out of 120) failed to adequately assess the assumption of stationarity^2^ in the observed time series. Among these 60 studies, 28 (46.7%) relied on the lag‐1 autoregressive (AR(1)) model and its variations, in ways that assume stationarity of the time‐series. The stationary AR(1) model posits that the outcome variable at a given time point ($y_{t}$) is linearly related to its value at the previous time point ($y_{t-1}$). This widespread reliance on the stationary AR(1) model without proper assessment of stationarity in the observed time‐series raises concerns regarding the validity of the findings.

On a positive note, psychological methodologists have made significant progress extending the AR(1) model and developing a wide range of approaches to account for nonstationarity in intensive longitudinal data (see Table 1). These approaches have proven valuable for capturing changing temporal dynamics. Nonetheless, with them comes the challenge of determining which model is most suitable for describing the temporal dynamics. This boils down to *model selection*: choosing the best model among a set of candidate models (e.g., Bringmann et al., 2017; Hamaker et al., 2016, 2009). To find the model that describes the data‐generating mechanism most accurately, in‐sample information criteria (IC) are often used (Ding et al., 2018). On the other hand, when the goal of model selection is to find the model with optimal predictive accuracy, researchers can use out‐of‐sample model selection approaches such as cross‐validation and validation with a separate unseen dataset (e.g., Bulteel et al., 2018; Lafit et al., 2022; Liu & Zhou, 2023; Revol et al., 2024).

**TABLE 1:** An overview of univariate nonstationary models based on the AR(1) model for intensive longitudinal data analysis.

Types of nonstationarity	Description	Models	Example applications
Deterministic trend	A (linear) trend of time	AR(1) model with trend	Bak et al. (2016), Ernst et al. (2020), Frumkin et al. (2020), McNeish and Hamaker (2020) and Piccirillo and Rodebaugh (2022)
Stochastic trend	The first‐order difference of a time‐series is stationary	Unit root process (random walk)	Cerqueira et al. (2020) and Ratcliff (1978)
Known structural breaks	A change of parameters at a known time‐point	Change‐point model (Hamilton, 1989); moderated AR model (Adolf et al., 2017)	Albers and Bringmann (2020), Cabrieto et al. (2018)
Unknown structural breaks	A change of parameters at unknown time‐points	Hidden Markov model (Rabiner, 1989); regime‐switching AR(1) model (Kim & Halbert, 1999); threshold AR model (Tong & Lim, 1980)	Berkhout et al. (2023), Hamaker et al. (2016), Ji et al. (2020) and Madhyastha et al. (2011)
Gradual change	A smooth change of parameters over time	Time‐varying parameter modelled with the generalized additive model (Bringmann et al., 2017) or kernel‐smoothing (Haslbeck et al., 2021)	Helmich et al. (2023), Nemesure et al. (2024) and Siepe et al. (2024)

Despite the extensive work on model selection for stationary time‐series (e.g. determining the lag order of a stationary autoregressive process), how accurately different model selection approaches can identify the correct nonstationary process of a time‐series has remained largely unexplored (Kapetanios, 2001; Kim, 2012). This is a more challenging task than selecting among stationary models, which usually only involves selecting among nested models that differ in the number of covariates. Yet the candidate nonstationary models that describe different processes differ largely in their specifications and are thus sometimes non‐nested models. To improve the rigor of model selection in the analysis of nonstationary time‐series, Hamaker et al. (2016) argues that it is imperative to first investigate through simulations how the commonly used model selection techniques perform in selecting among nonstationary time‐series models.

In the remainder of the paper, we first describe the commonly used (non)stationary time‐series models and model selection techniques. Next, we conduct a simulation study to evaluate the performance of various model selection approaches when the candidate models differ in the type of nonstationarity that they accommodate. Additionally, we explore how their performances are influenced by three characteristics of the time‐series data: (1) the number of time‐points, (2) the average autocorrelation and (3) the type and extent of nonstationarity. Applying the findings from our simulation study, we also present a re‐analysis of an empirical dataset of nonstationary time‐series (Kossakowski et al., 2017), where researchers have demonstrated the usage of different nonstationary models (Albers & Bringmann, 2020; Cabrieto et al., 2018; Haslbeck et al., 2021). From this re‐analysis, we wish to (1) demonstrate our recommended workflow of analysing nonstationary time‐series data and (2) compare our model selection results with the modeling choices of previous studies on this dataset.

## 2 UNIVARIATE TIME‐SERIES MODELS

In the current study, we limit our scope to univariate models based on the AR(1) model that can generate stationary or nonstationary time‐series. We start from the basic stationary AR(1) model and move on to six common extensions that can generate nonstationary time‐series: random walk, AR(1) model with deterministic trend, time‐varying AR(1) model, threshold AR(1) model, hidden Markov model and regime‐switching AR(1) model. Here, we provide an overview of these models (for a more detailed description of each model, see Appendix S1: A).

### 2.1 AR(1) model

The stationary AR(1) model is widely used to describe psychological dynamic processes and can be written as:

(1) $$y_{t}=\alpha +\phi y_{t-1}+\epsilon_{t},$$

with α being the intercept, ϕ the autoregressive effect (−1<ϕ<1) and $\epsilon_{t}$ the independent and identically normally distributed innovation (or error) term which represents random fluctuations in the time‐series. Particularly, researchers are interested in the autoregressive effect, which reflects the inertia (resistance to change) of the variable of interest (Kuppens et al., 2010). The special case of ϕ=0 is called a *white noise* model since there is no temporal dependency in the time‐series.

### 2.2 Random walk

Random walk is a special case of the AR(1) model where ϕ=1. Because of its simplicity, researchers often use the random walk as a benchmark model in evaluating the performance of more complex time‐series models (Cerqueira et al., 2020; Kwas & Rubaszek, 2021).

Next, we present a few nonstationary extensions of the AR(1) model that allow the intercept or/and the autoregressive effect to vary over time.

### 2.3 AR(1) model with deterministic trends

By adding a trend to an AR(1) model, increases or decreases in the mean of a time‐series can be accommodated (Ernst et al., 2020; McNeish & Hamaker, 2020; Piccirillo & Rodebaugh, 2022). Compared to the AR(1) model, this model includes an additional (usually linear) function of time (βt), which helps capture the trend in the data and avoid getting a biased estimate of the inertia (ϕ) in case of mean‐nonstationarity:

(2) $$y_{t}=\alpha +\beta t+\phi y_{t-1}+\epsilon_{t}.$$

### 2.4 Time‐varying AR(1) model

Nonstationarity due to gradual changes in the parameters of an AR(1) model can be accommodated through the *time‐varying AR(1) model* (TV‐AR(1); Bringmann et al., 2017; Haslbeck et al., 2021; Helmich et al., 2023; Nemesure et al., 2024; Siepe et al., 2024):

(3) $$y_{t}=\alpha_{t}+\phi_{t}y_{t-1}+\epsilon_{t}.$$

This model is used to describe processes where both α and ϕ vary over time in a continuous manner, and is thus appropriate for describing nonstationarity due to slowly changing dynamics.

### 2.5 Threshold AR(1) model

If a time‐series contains multiple regimes with different temporal dynamics, one relevant model is the threshold AR(1) model (Hamaker et al., 2009; Madhyastha et al., 2011; Tong & Lim, 1980). This model represents a process where the time‐series data is generated by distinct AR(1) models at different time‐points, depending on the value of a threshold variable. We study the simple case of *self‐exciting threshold autoregressive model*, or SETAR(2,1,1):

(4) $$y_{t}=\left\{\begin{array}{cc} \alpha^{\left(1\right)}+\phi^{\left(1\right)}y_{t-1}+\epsilon_{t}^{\left(1\right)},\; & \text{if}\;y_{t-1}\leq \tau \\ \alpha^{\left(2\right)}+\phi^{\left(2\right)}y_{t-1}+\epsilon_{t}^{\left(2\right)},\; & \text{if}\;y_{t-1}>\tau \end{array}\right.$$

In this model, the lagged outcome variable ($y_{t-1}$) is the threshold variable. Observations are generated by two different AR(1) models, depending on whether their lag‐1 values are above or below a threshold value.

### 2.6 Hidden Markov model and regime‐switching AR(1) model

Alternatively, multiple regimes in a time‐series can be modeled based on the Markov Chain. These models do not consider that the regime‐switching relies on a threshold variable as in the threshold AR(1) model. Instead, they consider the current regime at each time‐point as a random event: each regime has a prior probability of persisting or switching to another regime (Hamaker et al., 2016). In this study, we consider two models that allow Markov Chain‐based regime‐switching: (1) *a hidden Markov model —*(HMM; Choi‐Kain et al., 2022; Stifter & Rovine, 2015), which describes each regime as a white noise process with different means, and (2) the *regime‐switching AR(1) model* (RS‐AR(1); Hamaker et al., 2016; Ji et al., 2020), which describes each regime as a different AR(1) model.

## 3 MODEL SELECTION TECHNIQUES

In the previous section, we presented various models that can describe different types of nonstationarity. When lacking a theoretical understanding of an observed time‐series, empirical researchers can fit multiple candidate models to the data and conduct *model selection* to decide on an “optimal” model. Here, we briefly review important concepts related to the task of model selection and commonly used techniques.

### 3.1 Model selection: what for?

Time‐series analysis conducted in today's psychology research typically prioritizes explanation (i.e. describing the data‐generating mechanism) over prediction (Yarkoni & Westfall, 2017): in our systematic review (https://osf.io/ahj3u), only 18 out of 120 studies (15%) assessed the predictive accuracy of models. However, more recently researchers have argued that both explanatory and predictive capabilities are important to consider when conducting model selection (Hofman et al., 2021; Shmueli, 2010).

In the dimension of explanation, the optimal model should reflect the correct data‐generating mechanism — the “true” model. However, assuming the candidate models include the true model can be unrealistic since the reality is usually more complicated than any parametric model. With the belief of “all models are wrong”, Box (1976) argued that “simple but evocative” models should be pursued as useful approximations of the true model.

When evaluating the predictive capability of a model, the optimal model should make the most accurate predictions for unseen observations (Ding et al., 2018). Prediction‐focused model selection does not assume the existence of a true model, instead uses predictive accuracy as the sole criterion. Achieving optimal predictive accuracy requires properly balancing overfitting and underfitting^3^ (Yarkoni & Westfall, 2017). The selected model should be neither too complex and thus driven by noise in the sample nor too simple to capture the signal sufficiently (Claeskens & Hjort, 2008). Therefore, parsimony is an important guiding principle for both prediction‐focused and explanation‐focused model selection. In the simulation study, we will apply model selection techniques for both purposes.

### 3.2 Explanation‐focused: information criteria

When the goal of model selection is to find the best‐fitting model, it is imperative to quantify how similar a candidate model is to the true model. For this purpose, Kullback and Leibler (1951) discovered the Kullback‐Leibler (KL) discrepancy, which is well suited to quantify such similarity and forms the basis of many commonly used model selection criteria, as further discussed in Appendix S1: B.

Akaike Information Criteria (AIC; Akaike, 1974) was then developed and intended as an asymptotically^4^ unbiased estimator of the K‐L discrepancy between the distributions generated by the true model and the candidate model:

(5) $$\begin{array}{ll} \text{AIC} & =-2(\text{log}(ℒ)-p)\\ & =-2\text{log}(ℒ)+2p,\; \end{array}$$

where ℒ denotes the estimated likelihood of a candidate model, and p the number of freely estimated parameters in the model. AIC measures the “badness of fit” of a model by penalizing model complexity (i.e. deducting p from the log‐likelihood) and assigning a negative weight (i.e. −2) to the result. Despite its ease of use, AIC has shown two limitations in choosing the correct order of an autoregressive model depending on the sample size: (1) a tendency to select overspecified models (i.e. more complex than the true model) in small samples (Hurvich & Tsai, 1989); and (2) an inability to select the true model consistently in large samples (Shibata, 1976). In response to limitation (1), Hurvich and Tsai (1989), Sugiura (1978) developed AICc, which further penalizes model complexity to correct AIC's small‐sample bias. In the context of single‐case (N=1) time‐series analysis, $\text{AI}{\text{C}}_{c}$ can be expressed as (Hurvich & Tsai 1989):

(6) $$\text{AI}{\text{C}}_{c}=\text{AIC}+\frac{2p(p+1)}{T-p-1},$$

with T denoting the number of predictable time‐points.^5^

To tackle challenge (2), adaptations of AIC were developed and showed consistency (Claeskens & Hjort, 2008), of which the most commonly used are the Bayesian Information Criteria (BIC; Schwarz, 1978) and the Hannan‐Quinn Criteria (HQ; Hannan & Quinn, 1979):

(7) BIC=−2log(ℒ)+log(T)p,

(8) HQ=−2log(ℒ)+2log(log(T))p.

The BIC assigns a heavier weight (log(T)) to penalize model complexity than the AIC and is thus less likely to overfit in small samples (and can even underfit). The HQ only applies a slightly stronger penalty to model complexity than the AIC with realistic sample size.^6^ Therefore, the HQ has similar asymptotic behaviors to the BIC, but performs similarly to the AIC in small samples.

All the aforementioned information criteria (IC) rely on calculating a model's likelihood, typically assuming normal, independent and identically distributed (i.i.d.) errors. However, these assumptions may not hold in empirical data, raising concerns about the appropriateness of using such IC in these cases. Therefore, model selection techniques with fewer assumptions are particularly valuable when assumption violations occur.

### 3.3 Prediction‐focused: cross‐validation

The goal of prediction‐focused model selection is minimizing prediction error for unseen observations, which does not rely on any assumption of the particular error distribution. For an unseen observation that is independent of the data used for model estimation, $y_{t}$, we aim to minimize the *mean squared prediction error* (MSPE; Lafit et al., 2022; McQuarrie & Tsai, 1998):

(9) $$\text{MSPE}=E[{\left(y_{t}-{ŷ}_{t}\right)}^{2}],$$

where ${ŷ}_{t}$ is the predicted value by the model. To estimate the MSPE, a common approach is cross‐validation (CV). CV involves repeatedly dividing a sample into two parts: a training set to estimate a model and a validation set to evaluate the model's predictive accuracy. CV techniques differ in how they create the validation set. In the current study, we consider two techniques used by Bulteel et al. (2018): leave‐one‐out cross‐validation (LOO‐CV) and blocked cross‐validation (blocked‐CV).

**FIGURE 1:**
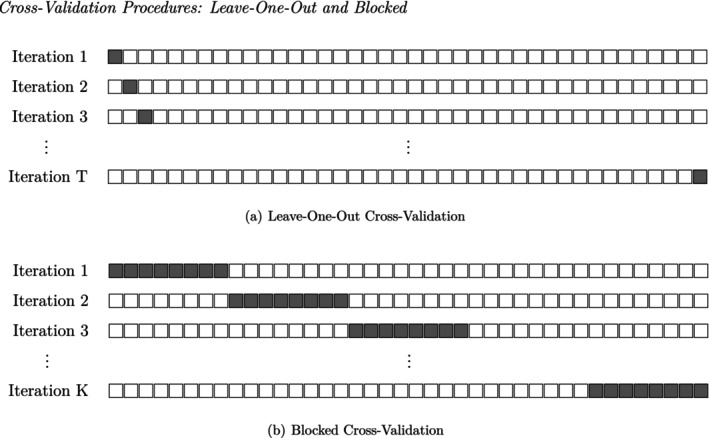
Cross‐validation procedures: Leave‐one‐out and blocked. For a dataset of T=40 observations, the figure represents the elements of the test set as dark‐colored squares and the elements of the training set as blank squares. Each iteration consists of T time‐points, with T total iterations in LOO‐CV and K in blocked CV.

Allen (1974) proposed LOO‐CV: each predictable observation in the time‐series, $y_{t}$ (t=2,3,…,T), is used as the validation set in one iteration, which repeats until exhaustion (as shown in Figure 1a). The prediction errors of all observations are then averaged to calculate the CV criterion, CV(1):

(10) $$\text{CV}{\left(1\right)}_{\text{LOO}}=\frac{1}{T}\overset{T}{\underset{i=1}{\sum }}{\left(y_{i}-{ŷ}_{i}\right)}^{2}.$$

Blocked‐CV works similarly to LOO‐CV, only that instead of one observation, T/k consecutive observations are used as the validation set (Snijders, 1988). As an example (visualized in Figure 1b), in 5‐block CV, the time‐series is first cut to five blocks of equal length. In each iteration, one block is held out for validation while the model is estimated on the remaining four blocks. The blocked‐CV criterion is calculated similarly to $\text{CV}{\left(1\right)}_{\text{LOO}}$. For a k‐blocked‐CV:

(11) $$\text{CV}{\left(1\right)}_{\text{Blocked}}=\frac{1}{T}\overset{K}{\underset{k=1}{\sum }}\overset{T^{\left(k\right)}}{\underset{i=1}{\sum }}{\left(y_{ik}-{ŷ}_{ik}\right)}^{2}.$$

Although CV and IC focus on different aspects of a model, they are intrinsically connected: AIC and BIC are asymptotically equivalent to LOO‐CV (Stone, 1977) and leave‐d‐out CV (Shao, 1997), where $d=T(1-\frac{1}{log(T)-1})$, respectively. Thus, for long time‐series, computationally expensive CV and computationally simple IC will converge to similar solutions (in certain situations already for a sample size of 40; Shao, 1997).

In a cross‐sectional setting, Wager (2020) showed that CV is asymptotically consistent in identifying the more accurate model. Whereas in small samples, CV tends to favor simple models as it overestimates the predictive accuracy of simple models and underestimates that of complex models (Liu & Zhou, 2023). In the current study, we examine whether the sample size dependence of CV's model preference also holds in a nonstationary setting.

### 3.4 Prediction‐focused: out‐of‐sample (OOS) predictive accuracy

A common concern of CV in time‐series analysis is that the training and validation sets are not independent due to the temporal dependency in data (Rabinowicz & Rosset, 2022). Such assumption violation of CV can lead to bias in estimating MSPE and further result in incorrect model selection (McQuarrie & Tsai, 1998). To avoid this bias, we can directly calculate different models' MSPE on a simulated “population” dataset (Lafit et al., 2022; Revol et al., 2024) and get a definite answer on which model has the optimal predictive accuracy. This approach assumes that the true data‐generating model is known, which can hold in a simulation setting but not with empirical data. Therefore, this technique is not intended for model selection with empirical data.

To conduct OOS prediction, we simulate a long time‐series (e.g. T=100,000) based on the true model, separately and independently from the shorter training samples. This long time‐series is considered the “population” and used for calculating the MSPE of different models for selection in a completely out‐of‐sample manner.

## 4 SIMULATION STUDY

The goal of this simulation study is to evaluate the performance of the aforementioned model selection techniques when applying them to nonstationary time‐series. In this study, we first simulate univariate time‐series data following all the models described earlier: (1) the stationary AR(1) model (including white noise as a special case), (2) the random walk model, (3) the AR(1) model with linear time trend, (4) the TV‐AR(1) model (including two cases: intercept or autocorrelation varies), (5) the SETAR(2,1,1) model, (6) the HMM and (7) the RS‐AR(1) model. Then, we fit these nine candidate models to the simulated time‐series and conducted model selection using IC (AIC, AICc, BIC, HQ), CV (LOO‐CV, 10‐block CV) and out‐of‐sample (OOS) predictive accuracy.

With the simulation study, we aim to answer two main research questions: (1) how frequently do model selection techniques identify the true model; and (2) which model has the optimal predictive accuracy if not the true model?

### 4.1 Simulation Design

Given each data‐generating model, we first specified 31 simulation conditions that vary on three factors: the sample size (the number of time‐points), the autocorrelation and the extent of nonstationarity.^7^ Four sample sizes (T=50,100,200,1000) were used for data generation in each simulation condition. This range is consistent with similar studies (e.g., Bringmann et al., 2017; Hamaker et al., 2009) and contains small yet commonly used sample sizes and very large sample sizes. This allows us to investigate both the small‐sample (practical) and asymptotic (ideal) performance of the model selection techniques. The autocorrelation and extent of nonstationarity in all simulation conditions are summarized in Table 2. We simulated 100 time‐series for each sample size across all simulation conditions (same as Haslbeck et al., 2021). To briefly assess the robustness of model selection techniques against assumption violation, we specified two additional simulation conditions with non‐normal innovations using the sample size of T=100. In these conditions, innovations follow a skewed normal distribution with location parameter ξ=0, scale parameter ω=2 and shape parameter α=40 to resemble the positively skewed distribution of negative affect, which is commonly observed in time‐series data (Haslbeck et al., 2023). The two conditions are based on the AR(1) model ($y_{t}=.3y_{t-1}+\epsilon_{t}$) and the AR(1) with linear trend model ($y_{t}=.1t+.3y_{t-1}+\epsilon_{t}$).

**TABLE 2:** Simulation conditions used in the study (excluding time‐points).

Model	Specification
AR(1)	$y_{t}=\epsilon_{t}$ (White noise)
	$y_{t}=.3y_{t-1}+\epsilon_{t}$ ^a^
	$y_{t}=.7y_{t-1}+\epsilon_{t}$
Random walk	$y_{t}=y_{t-1}+\epsilon_{t}$
AR(1) with linear trend	$y_{t}=.1t+.3y_{t-1}+\epsilon_{t}$ ^a^
	$y_{t}=.5t+.3y_{t-1}+\epsilon_{t}$
	$y_{t}=.1t+.7y_{t-1}+\epsilon_{t}$
	$y_{t}=.5t+.7y_{t-1}+\epsilon_{t}$
TV‐AR(1) with intercept change	$y_{t}=.5\sin\frac{2\pi t}{T}+.3y_{t-1}+\epsilon_{t}$
	$y_{t}=2.5\sin\frac{2\pi t}{T}+.3y_{t-1}+\epsilon_{t}$
	$y_{t}=.5\sin\frac{2\pi t}{T}+.7y_{t-1}+\epsilon_{t}$
	$y_{t}=2.5\sin\frac{2\pi t}{T}+.7y_{t-1}+\epsilon_{t}$
TV‐AR(1) with autocorrelation change	$y_{t}=(.05\sin\frac{2\pi t}{T}+.3)y_{t-1}+\epsilon_{t}$
	$y_{t}=(.25\sin\frac{2\pi t}{T}+.3)y_{t-1}+\epsilon_{t}$
	$y_{t}=(.05\sin\frac{2\pi t}{T}+.7)y_{t-1}+\epsilon_{t}$
	$y_{t}=(.25\sin\frac{2\pi t}{T}+.7)y_{t-1}+\epsilon_{t}$
SETAR(2,1,1)	$y_{t}=\left\{\begin{array}{cc} 1+.4y_{t-1}+\epsilon_{t},\; & \text{if}\;y_{t-1}\leq 3\\ 2+.4y_{t-1}+\epsilon_{t},\; & \text{if}\;y_{t-1}>3 \end{array}\right.$
	$y_{t}=\left\{\begin{array}{cc} 1+.8y_{t-1}+\epsilon_{t},\; & \text{if}\;y_{t-1}\leq 3\\ 1+.4y_{t-1}+\epsilon_{t},\; & \text{if}\;y_{t-1}>3 \end{array}\right.$
	$y_{t}=\left\{\begin{array}{cc} 1+.8y_{t-1}+\epsilon_{t},\; & \text{if}\;y_{t-1}\leq 3\\ 2+.4y_{t-1}+\epsilon_{t},\; & \text{if}\;y_{t-1}>3 \end{array}\right.$
HMM	$y_{t}=\left\{\begin{array}{cc} -.5+\epsilon_{t},\; & \text{if}\;s_{t}=1\\ .5+\epsilon_{t},\; & \text{if}\;s_{t}=2 \end{array}\right.$, switches at $\frac{T}{2}$
	$y_{t}=\left\{\begin{array}{cc} -2.5+\epsilon_{t},\; & \text{if}\;s_{t}=1\\ 2.5+\epsilon_{t},\; & \text{if}\;s_{t}=2 \end{array}\right.$, switches at $\frac{T}{2}$
	$y_{t}=\left\{\begin{array}{cc} -.5+\epsilon_{t},\; & \text{if}\;s_{t}=1\\ .5+\epsilon_{t},\; & \text{if}\;s_{t}=2 \end{array}\right.$, switches at $\frac{T}{4}$, $\frac{T}{2}$ and $\frac{3T}{4}$
	$y_{t}=\left\{\begin{array}{cc} -2.5+\epsilon_{t},\; & \text{if}\;s_{t}=1\\ 2.5+\epsilon_{t},\; & \text{if}\;s_{t}=2 \end{array}\right.$, switches at $\frac{T}{4}$, $\frac{T}{2}$ and $\frac{3T}{4}$
RS‐AR(1)	$y_{t}=\left\{\begin{array}{cc} .25y_{t-1}+\epsilon_{t},\; & \text{if}\;s_{t}=1\\ .35y_{t-1}+\epsilon_{t},\; & \text{if}\;s_{t}=2 \end{array}\right.$, switches at $\frac{T}{2}$
	$y_{t}=\left\{\begin{array}{cc} .05y_{t-1}+\epsilon_{t},\; & \text{if}\;s_{t}=1\\ .55y_{t-1}+\epsilon_{t},\; & \text{if}\;s_{t}=2 \end{array}\right.$, switches at $\frac{T}{2}$
	$y_{t}=\left\{\begin{array}{cc} .65y_{t-1}+\epsilon_{t},\; & \text{if}\;s_{t}=1\\ .75y_{t-1}+\epsilon_{t},\; & \text{if}\;s_{t}=2 \end{array}\right.$, switches at $\frac{T}{2}$
	$y_{t}=\left\{\begin{array}{cc} .45y_{t-1}+\epsilon_{t},\; & \text{if}\;s_{t}=1\\ .95y_{t-1}+\epsilon_{t},\; & \text{if}\;s_{t}=2 \end{array}\right.$, switches at $\frac{T}{2}$
	$y_{t}=\left\{\begin{array}{cc} .25y_{t-1}+\epsilon_{t},\; & \text{if}\;s_{t}=1\\ .35y_{t-1}+\epsilon_{t},\; & \text{if}\;s_{t}=2 \end{array}\right.$, switches at $\frac{T}{4}$, $\frac{T}{2}$ and $\frac{3T}{4}$
	$y_{t}=\left\{\begin{array}{cc} .05y_{t-1}+\epsilon_{t},\; & \text{if}\;s_{t}=1\\ .55y_{t-1}+\epsilon_{t},\; & \text{if}\;s_{t}=2 \end{array}\right.$, switches at $\frac{T}{4}$, $\frac{T}{2}$ and $\frac{3T}{4}$
	$y_{t}=\left\{\begin{array}{cc} .65y_{t-1}+\epsilon_{t},\; & \text{if}\;s_{t}=1\\ .75y_{t-1}+\epsilon_{t},\; & \text{if}\;s_{t}=2 \end{array}\right.$, switches at $\frac{T}{4}$, $\frac{T}{2}$ and $\frac{3T}{4}$
	$y_{t}=\left\{\begin{array}{cc} .45y_{t-1}+\epsilon_{t},\; & \text{if}\;s_{t}=1\\ .95y_{t-1}+\epsilon_{t},\; & \text{if}\;s_{t}=2 \end{array}\right.$, switches at $\frac{T}{4}$, $\frac{T}{2}$ and $\frac{3T}{4}$

*Note*: For all conditions, the innovation follows a normal distribution of 𝒩(0,1). For all conditions where the data‐generating model is either HMM or RS‐AR(1), the simulated time‐series begins at regime 1 ($s_{1}=1$).

^a^ For each of these two model specifications, a condition with the innovation following a skewed normal distribution is simulated.

In each iteration, for all models except for the Hidden Markov model and the regime‐switching AR(1) model, we estimated them by directly regressing $y_{t=2}$, $y_{t=3}$, …, $y_{t=101}$ on $y_{t=1}$, $y_{t=2}$, …, $y_{t=100}$. Hence, we did not employ the state‐space framework for estimation, which allows, for instance, incorporating measurement error in the estimation (Durbin & Koopman, 2012). Further details of model estimation and selection are summarized in Appendix S1: C.

### 4.2 Results

In this section, we first discuss convergence issues when estimating the HMM and the RS‐AR(1) model. Then, for each data‐generating model, we evaluate the performance of all model selection techniques on how frequently each technique identifies the true model, and which model has the optimal predictive accuracy. We describe in detail the findings of simulations using the AR(1) model with and without a linear trend as the data‐generating process. We further summarize the main findings of the simulation study here and provide the results of other simulation conditions in Appendix S1: D.

#### 4.2.1 Convergence of the HMM and the RS‐AR(1) model

As discussed earlier, the estimation of the HMM and the RS‐AR(1) model relies on the state‐space model framework. This leads to the occasional non‐convergence of these two models in the simulation study. In the simulation conditions where data are generated with a linear trend of time, estimations of both models often fail to converge, especially when the trend or the sample size is large. For random walk time‐series with 1000 time‐points, estimations of both models also fail to converge half of the time. Such non‐convergence is not too concerning as empirical researchers can usually identify linear trend and random walk through visual inspection and statistical tests.

#### 4.2.2 True model being the AR(1) model

Table 3 presents results for the three simulation conditions where the true model is the stationary AR(1) model with normal innovations. Conditions where the true models are parsimonious (i.e. AR(1), white noise and random walk) provide valuable insight into whether model selection techniques tend to select overspecified models. Overall, BIC and OOS predictive accuracy perform the best in retrieving the true model (i.e. each having the highest average rank in 50% of the simulation conditions). Particularly, the performance of BIC improves as T becomes larger. The performance of LOOCV and 10‐block CV is also satisfactory, as the “optimal” model they identify after 100 iterations is correct in all simulation conditions. The other three model selection techniques, AIC, AICc and HQ, all show a tendency to overfit in certain circumstances. AIC constantly prefers a more complex model (SETAR). AICc is less likely to overfit than AIC in small samples, but overfits when T gets larger. HQ overfits in small samples (similar to AIC as discussed earlier) but identifies the true model in larger samples.

**TABLE 3:** Performance of model selection techniques when the true model is stationary AR(1) model.

Condition	*T*	Criteria	AIC	AICc	HQ	BIC	LOOCV	Blocked CV	OOS prediction
ϕ=.7	50	Rank	2.91 (.11)	2.20 (.11)	2.70 (.10)	2.24 (.08)	2.12 (.10)	1.86 (.09)	**1.38** (.06)
		Proportion	.07 (.03)	.30 (.05)	.06 (.02)	.12 (.03)	.33 (.05)	.42 (.05)	.70 (.05)
		Optimal	SETAR (1.89)	AR(1) (2.20)	SETAR (1.34)	SETAR (1.43)	AR(1) (2.12)	AR(1) (1.86)	AR(1) (1.38)
	100	Rank	2.88 (.12)	2.52 (.12)	2.23 (.09)	1.63 (.07)	2.04 (.10)	1.87 (.09)	**1.30** (.05)
		Proportion	.05 (.02)	.17 (.04)	.16 (.04)	.48 (.05)	.35 (.05)	.38 (.05)	.72 (.04)
		Optimal	SETAR (2.05)	AR(1) (2.52)	SETAR (1.67)	AR(1) (1.63)	AR(1) (2.04)	AR(1) (1.87)	AR(1) (1.30)
	200	Rank	2.40 (.11)	2.28 (.10)	1.63 (.07)	**1.24** (.05)	1.89 (.10)	1.78 (.09)	1.31 (.07)
		Proportion	.21 (.04)	.25 (.04)	.45 (.05)	.78 (.04)	.46 (.05)	.47 (.05)	.78 (.04)
		Optimal	SETAR (2.01)	AR(1) (2.28)	AR(1) (1.63)	AR(1) (1.24)	AR(1) (1.89)	AR(1) (1.78)	AR(1) (1.31)
	1000	Rank	2.46 (.12)	2.43 (.12)	1.39 (.07)	**1.06** (.02)	NA (NA)	NA (NA)	1.73 (.09)
		Proportion	.18 (.04)	.19 (.04)	.69 (.05)	.94 (.02)	NA (NA)	NA (NA)	.53 (.05)
		Optimal	SETAR (1.85)	SETAR (1.91)	AR(1) (1.39)	AR(1) (1.06)	NA (NA)	NA (NA)	AR(1) (1.73)
ϕ=.3	50	Rank	3.28 (.13)	2.48 (.13)	2.90 (.10)	2.50 (.09)	2.58 (.12)	2.56 (.14)	**1.67** (.09)
		Proportion	.03 (.02)	.26 (.04)	.02 (.01)	.10 (.03)	.22 (.04)	.28 (.04)	.57 (.05)
		Optimal	SETAR (1.94)	AR(1) (2.48)	SETAR (1.34)	SETAR (1.63)	AR(1) (2.58)	AR(1) (2.56)	AR(1) (1.67)
	100	Rank	2.94 (.13)	2.66 (.13)	2.34 (.11)	1.89 (.09)	2.26 (.10)	2.16 (.11)	**1.46** (.08)
		Proportion	.11 (.03)	.21 (.04)	.19 (.04)	.39 (.05)	.27 (.04)	.32 (.05)	.68 (.05)
		Optimal	SETAR (1.87)	SETAR (2.58)	SETAR (1.57)	AR(1) (1.89)	AR(1) (2.26)	AR(1) (2.16)	AR(1) (1.46)
	200	Rank	2.61 (.11)	2.43 (.11)	1.87 (.09)	1.28 (.05)	1.91 (.10)	1.98 (.10)	**1.24** (.05)
		Proportion	.13 (.03)	.19 (.04)	.38 (.05)	.75 (.04)	.42 (.05)	.41 (.05)	.80 (.04)
		Optimal	SETAR (1.91)	SETAR (2.27)	AR(1) (1.87)	AR(1) (1.28)	AR(1) (1.91)	AR(1) (1.98)	AR(1) (1.24)
	1000	Rank	2.61 (.10)	2.60 (.10)	1.47 (.07)	**1.11** (.04)	NA (NA)	NA (NA)	1.80 (.10)
		Proportion	.10 (.03)	.10 (.03)	.63 (.05)	.92 (.03)	NA (NA)	NA (NA)	.50 (.05)
		Optimal	SETAR (1.73)	SETAR (1.79)	AR(1) (1.47)	AR(1) (1.11)	NA (NA)	NA (NA)	AR(1) (1.80)
ϕ=0 (White noise)	50	Rank	2.74 (.16)	1.79 (.14)	2.23 (.12)	1.66 (.09)	2.18 (.14)	2.13 (.15)	**1.63** (.09)
		Proportion	.14 (.03)	.62 (.05)	.18 (.04)	.49 (.05)	.38 (.05)	.42 (.05)	.57 (.05)
		Optimal	SETAR (1.77)	WN (1.79)	SETAR (1.32)	WN (1.66)	WN (2.18)	WN (2.13)	WN (1.63)
	100	Rank	2.44 (.15)	2.03 (.14)	1.72 (.10)	**1.22** (.06)	2.18 (.16)	2.23 (.14)	1.50 (.07)
		Proportion	.28 (.04)	.49 (.05)	.49 (.05)	.83 (.04)	.42 (.05)	.37 (.05)	.58 (.05)
		Optimal	SETAR (2.29)	WN (2.03)	WN (1.72)	WN (1.22)	WN (2.18)	WN (2.23)	WN (1.50)
	200	Rank	2.72 (.16)	2.50 (.15)	1.55 (.08)	**1.09** (.03)	2.32 (.14)	2.16 (.12)	1.61 (.08)
		Proportion	.23 (.04)	.27 (.04)	.57 (.05)	.91 (.03)	.34 (.05)	.37 (.05)	.55 (.05)
		Optimal	SETAR (1.99)	SETAR (2.33)	WN (1.55)	WN (1.09)	WN (2.32)	WN (2.16)	WN (1.61)
	1000	Rank	2.67 (.17)	2.63 (.17)	1.39 (.09)	**1.01** (.01)	NA (NA)	NA (NA)	1.53 (.08)
		Proportion	.26 (.04)	.27 (.04)	.75 (.04)	.99 (.01)	NA (NA)	NA (NA)	.60 (.05)
		Optimal	SETAR (2.28)	SETAR (2.36)	WN (1.39)	WN (1.01)	NA (NA)	NA (NA)	WN (1.53)

*Note*: “optimal”, the model with the highest average rank (its average rank); “proportion”, the proportion of correct model selection (standard error); “rank”, the average rank of the true data‐generating model (standard error). For each condition, the model selection technique with the best performance (having the highest average rank for the true model) is highlighted in bold.

Table 4 shows the performance of the model selection techniques when the true model is the AR(1) model with skewed innovations. Given the same autocorrelation and sample size (ϕ=.3, T=100), BIC performs less well in the current condition than when innovations are normally distributed, as indicated by the lower average rank of the AR(1) model estimates (2.74 vs. 1.89). However, the performance of both CV techniques and OOS prediction does not differ between the two conditions. Such findings confirm that likelihood‐based ICs do not perform well when model assumptions are not met.

**TABLE 4:** Performance of model selection techniques when the true model is an AR(1) model with innovations following a skewed normal distribution.

Condition	*T*	Criteria	AIC	AICc	HQ	BIC	LOOCV	Blocked CV	OOS prediction
ϕ=.3 with skewed innovations	100	Rank	4.38 (.14)	4.06 (.14)	3.61 (.14)	2.74 (.73)	2.16 (.13)	1.93 (.11)	**1.41** (.06)
		Proportion	.02 (.01)	.02 (.01)	.04 (.02)	.17 (.04)	.40 (.05)	.46 (.05)	.63 (.05)
		Optimal	RS‐AR(1) (2.04)	RS‐AR(1) (2.05)	SETAR (2.35)	SETAR (2.62)	AR(1) (2.16)	AR(1) (1.93)	AR(1) (1.41)

*Note*: Different from other tables in this section, “rank” refers to the average rank of the AR(1) model estimated with the OLS estimator assuming normally distributed innovations (standard error). For each condition, the model selection technique with the best performance (having the highest average rank for the true model) is highlighted in bold.

#### 4.2.3 True model being the AR(1) model with linear trend

Table 5 shows that most model selection techniques perform well on time‐series generated by the AR(1) model with linear trend and normal innovations, especially CV and out‐of‐sample prediction. AIC and AICc frequently select the AR(1) model with time‐varying intercept and are most likely to select overspecified models.^8^

**TABLE 5:** Performance of model selection techniques when the true model is AR(1) model with linear trend.

Condition	*T*	Criteria	AIC	AICc	HQ	BIC	LOOCV	Blocked CV	OOS prediction
ϕ=.3, β=.1	50	Rank	1.49 (.06)	1.40 (.05)	1.37 (.06)	**1.21** (.05)	1.28 (.05)	1.23 (.04)	1.30 (.05)
		Proportion	.54 (.05)	.62 (.05)	.67 (.05)	.81 (.04)	.74 (.04)	.78 (.04)	.72 (.04)
		Optimal	AR(1) with trend (1.49)	AR(1) with trend (1.40)	AR(1) with trend (1.37)	AR(1) with trend (1.21)	AR(1) with trend (1.28)	AR(1) with trend (1.23)	AR(1) with trend (1.30)
	100	Rank	1.57 (.05)	1.47 (.05)	1.32 (.05)	**1.05** (.02)	1.35 (.05)	1.18 (.04)	1.18 (.04)
		Proportion	.43 (.05)	.53 (.05)	.68 (.05)	.95 (.02)	.65 (.05)	.82 (.04)	.82 (.04)
		Optimal	TV‐AR(1): α varies (1.43)	AR(1) with trend (1.47)	AR(1) with trend (1.32)	AR(1) with trend (1.05)	AR(1) with trend (1.35)	AR(1) with trend (1.18)	AR(1) with trend (1.18)
	200	Rank	1.43 (.05)	1.41 (.05)	1.11 (.03)	**1.01** (.01)	1.25 (.04)	1.15 (.04)	1.30 (.05)
		Proportion	.57 (.05)	.59 (.05)	.89 (.03)	.99 (.01)	.75 (.04)	.85 (.04)	.70 (.05)
		Optimal	AR(1) with trend (1.43)	AR(1) with trend (1.41)	AR(1) with trend (1.11)	AR(1) with trend (1.01)	AR(1) with trend (1.25)	AR(1) with trend (1.15)	AR(1) with trend (1.30)
	1000	Rank	1.43 (.05)	1.42 (.05)	1.06 (.02)	**1.01** (.01)	NA (NA)	NA (NA)	1.45 (.05)
		Proportion	.57 (.05)	.58 (.05)	.94 (.02)	.99 (.01)	NA (NA)	NA (NA)	.55 (.05)
		Optimal	AR(1) with trend (1.43)	AR(1) with trend (1.42)	AR(1) with trend (1.06)	AR(1) with trend (1.01)	NA (NA)	NA (NA)	AR(1) with trend (1.45)
ϕ=.3, β=.5	50	Rank	1.60 (.05)	1.42 (.05)	1.34 (.05)	1.12 (.03)	1.27 (.04)	**1.10** (.03)	1.13 (.03)
		Proportion	.41 (.05)	.58 (.05)	.67 (.05)	.88 (.03)	.73 (.04)	.90 (.03)	.87 (.03)
		Optimal	TV‐AR(1): α varies (1.44)	AR(1) with trend (1.42)	AR(1) with trend (1.34)	AR(1) with trend (1.12)	AR(1) with trend (1.27)	AR(1) with trend (1.10)	AR(1) with trend (1.13)
	100	Rank	1.45 (.05)	1.40 (.05)	1.19 (.04)	**1.05** (.02)	1.25 (.04)	1.19 (.04)	1.25 (.04)
		Proportion	.55 (.05)	.60 (.05)	.81 (.04)	.95 (.02)	.75 (.04)	.81 (.04)	.75 (.04)
		Optimal	AR(1) with trend (1.45)	AR(1) with trend (1.40)	AR(1) with trend (1.19)	AR(1) with trend (1.05)	AR(1) with trend (1.25)	AR(1) with trend (1.19)	AR(1) with trend (1.25)
	200	Rank	1.40 (.05)	1.39 (.05)	1.11 (.03)	**1.01** (.01)	1.34 (.05)	1.22 (.04)	1.29 (.05)
		Proportion	.60 (.05)	.61 (.05)	.89 (.03)	.99 (.01)	.66 (.05)	.78 (.04)	.71 (.05)
		Optimal	AR(1) with trend (1.40)	AR(1) with trend (1.39)	AR(1) with trend (1.11)	AR(1) with trend (1.01)	AR(1) with trend (1.34)	AR(1) with trend (1.22)	AR(1) with trend (1.29)
	1000	Rank	1.40 (.05)	1.40 (.05)	1.07 (.03)	**1.00** (.00)	NA (NA)	NA (NA)	1.36 (.05)
		Proportion	.60 (.05)	.60 (.05)	.93 (.03)	1.00 (.00)	NA (NA)	NA (NA)	.64 (.05)
		Optimal	AR(1) with trend (1.40)	AR(1) with trend (1.40)	AR(1) with trend (1.07)	AR(1) with trend (1.00)	NA (NA)	NA (NA)	AR(1) with trend (1.36)
ϕ=.7, β=.1	50	Rank	2.02 (.08)	1.78 (.07)	2.14 (.11)	2.12 (.10)	1.52 (.06)	**1.50** (.11)	1.72 (.14)
		Proportion	.21 (.04)	.36 (.05)	.31 (.05)	.31 (.05)	.55 (.05)	.72 (.04)	.71 (.05)
		Optimal	TV‐AR(1): α varies (1.29)	TV‐AR(1): α varies (1.39)	TV‐AR(1): α varies (1.61)	RW (1.90)	AR(1) with trend (1.52)	AR(1) with trend (1.50)	AR(1) with trend (1.72)
	100	Rank	1.58 (.05)	1.57 (.05)	1.34 (.05)	**1.10** (.03)	1.38 (.05)	1.13 (.03)	1.26 (.07)
		Proportion	.42 (.05)	.44 (.05)	.67 (.05)	.90 (.03)	.62 (.05)	.87 (.03)	.85 (.04)
		Optimal	TV‐AR(1): α varies (1.44)	TV‐AR(1): α varies (1.44)	AR(1) with trend (1.34)	AR(1) with trend (1.10)	AR(1) with trend (1.38)	AR(1) with trend (1.13)	AR(1) with trend (1.26)
	200	Rank	1.56 (.05)	1.50 (.05)	1.14 (.03)	**1.01** (.01)	1.31 (.05)	1.12 (.03)	1.21 (.04)
		Proportion	.44 (.05)	.50 (.05)	.86 (.03)	.99 (.01)	.69 (.05)	.88 (.03)	.79 (.04)
		Optimal	TV‐AR(1): α varies (1.44)	AR(1) with trend (1.50)	AR(1) with trend (1.14)	AR(1) with trend (1.01)	AR(1) with trend (1.31)	AR(1) with trend (1.12)	AR(1) with trend (1.21)
	1000	Rank	1.39 (.05)	1.39 (.05)	1.05 (.02)	**1.00** (.00)	NA (NA)	NA (NA)	1.35 (.05)
		Proportion	.61 (.05)	.61 (.05)	.95 (.02)	1.00 (.00)	NA (NA)	NA (NA)	.65 (.05)
		Optimal	AR(1) with trend (1.39)	AR(1) with trend (1.39)	AR(1) with trend (1.05)	AR(1) with trend (1.00)	NA (NA)	NA (NA)	AR(1) with trend (1.35)
ϕ=.7, β=.5	50	Rank	1.91 (.06)	1.68 (.06)	1.79 (.08)	1.76 (.09)	1.38 (.05)	**1.21** (.06)	1.45 (.10)
		Proportion	.19 (.04)	.41 (.05)	.40 (.05)	.47 (.05)	.63 (.05)	.84 (.04)	.79 (.04)
		Optimal	TV‐AR(1): α varies (1.25)	TV‐AR(1): α varies (1.44)	TV‐AR(1): α varies (1.56)	AR(1) with trend (1.76)	AR(1) with trend (1.38)	AR(1) with trend (1.21)	AR(1) with trend (1.45)
	100	Rank	1.61 (.05)	1.56 (.05)	1.37 (.05)	1.13 (.04)	1.33 (.05)	**1.10** (.04)	1.18 (.05)
		Proportion	.39 (.05)	.44 (.05)	.63 (.05)	.88 (.03)	.67 (.05)	.92 (.03)	.86 (.03)
		Optimal	TV‐AR(1): α varies (1.39)	TV‐AR(1): α varies (1.44)	AR(1) with trend (1.37)	AR(1) with trend (1.13)	AR(1) with trend (1.33)	AR(1) with trend (1.10)	AR(1) with trend (1.18)
	200	Rank	1.43 (.05)	1.42 (.05)	1.19 (.04)	**1.01** (.01)	1.34 (.05)	1.14 (.03)	1.26 (.04)
		Proportion	.57 (.05)	.58 (.05)	.81 (.04)	.99 (.01)	.66 (.05)	.86 (.03)	.74 (.04)
		Optimal	AR(1) with trend (1.43)	AR(1) with trend (1.42)	AR(1) with trend (1.19)	AR(1) with trend (1.01)	AR(1) with trend (1.34)	AR(1) with trend (1.14)	AR(1) with trend (1.26)
	1000	Rank	1.45 (.05)	1.45 (.05)	1.08 (.03)	**1.00** (.00)	NA (NA)	NA (NA)	1.38 (.05)
		Proportion	.55 (.05)	.55 (.05)	.92 (.03)	1.00 (.00)	NA (NA)	NA (NA)	.62 (.05)
		Optimal	AR(1) with trend (1.45)	AR(1) with trend (1.45)	AR(1) with trend (1.08)	AR(1) with trend (1.00)	NA (NA)	NA (NA)	AR(1) with trend (1.38)

*Note*: For each condition, the model selection technique with the best performance (having the highest average rank for the true model) is highlighted in bold.

For the condition with skewed innovations, Table 6 suggests that the performance of likelihood‐based ICs is highly comparable to the condition with normal innovations. The ICs thus captured the strong signal induced by the linear trend in the data‐generating process well, despite assumption violation. Both CV techniques and OOS prediction also yield comparable results.

**TABLE 6:** Performance of model selection techniques when the true model is an AR(1) model with a linear trend and innovations following a skewed normal distribution.

Condition	*T*	Criteria	AIC	AICc	HQ	BIC	LOOCV	Blocked CV	OOS prediction
ϕ=.3,β=.1 with skewed innovations	100	Rank	1.52 (.05)	1.46 (.05)	1.22 (.04)	**1.03** (.02)	1.30 (.05)	1.21 (.04)	1.32 (.05)
		Proportion	.48 (.05)	.54 (.05)	.78 (.04)	.97 (.02)	.70 (.05)	.79 (.04)	.68 (.05)
		Optimal	TV‐AR(1): α varies (1.48)	AR(1) with trend (1.46)	AR(1) with trend (1.22)	AR(1) with trend (1.03)	AR(1) with trend (1.30)	AR(1) with trend (1.21)	AR(1) with trend (1.32)

*Note*: For each condition, the model selection technique with the best performance (having the highest average rank for the true model) is highlighted in bold.

#### 4.2.4 Summary of the findings

From the simulation study, we found that BIC often performs the best in selecting the correctly specified model with the sample size T≥100. Among other techniques, AIC, AICc and HQ tend to select overspecified models under certain conditions: AIC across all sample sizes, AICc in large samples and HQ in small samples. Lastly, blocked‐CV often selected under‐specified models and did not select the model with optimal predictive accuracy as indicated by the OOS prediction. Considering the good performance and low computational cost of using BIC, we recommend researchers to use it when conducting nonstationarity‐related model selection on empirical data. However, if severe assumption violation occurs in estimated models, we recommend researchers also use CV techniques as they do not impose strong assumptions on the distribution of the errors. Across the simulation conditions, it was easier to achieve correct model selection when the extent of nonstationarity (signal‐to‐noise ratio) and the sample size are large. Moreover, identifying the correct mechanism of autocorrelation change proved to be difficult in both the TV‐AR(1) model and the RS‐AR(1) model: none of the model selection criteria could recover the true model consistently.

## 5 EMPIRICAL EXAMPLE

**FIGURE 2:**
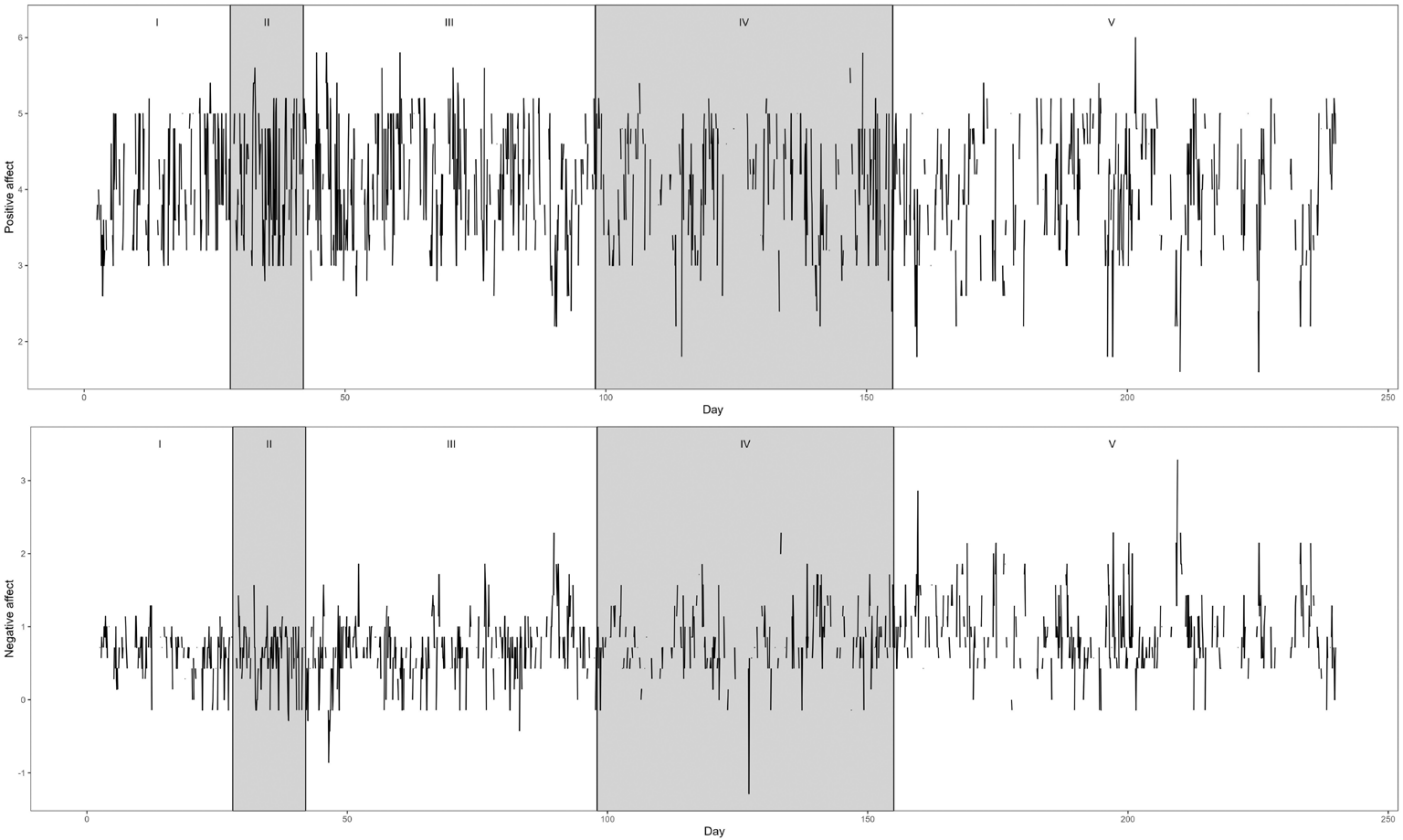
Time‐series of positive and negative affect in the study period. The time‐series can be separated into five phases: (I) baseline (day 1–28); (II) double‐blind period without antidepressant reduction (day 29–42); (III) double‐blind period with antidepressant reduction (day 43–98); (IV) planned post‐assessment after antidepressant reduction (day 99–155); (V) additional post‐assessment (day 156–239).

**FIGURE 3:**
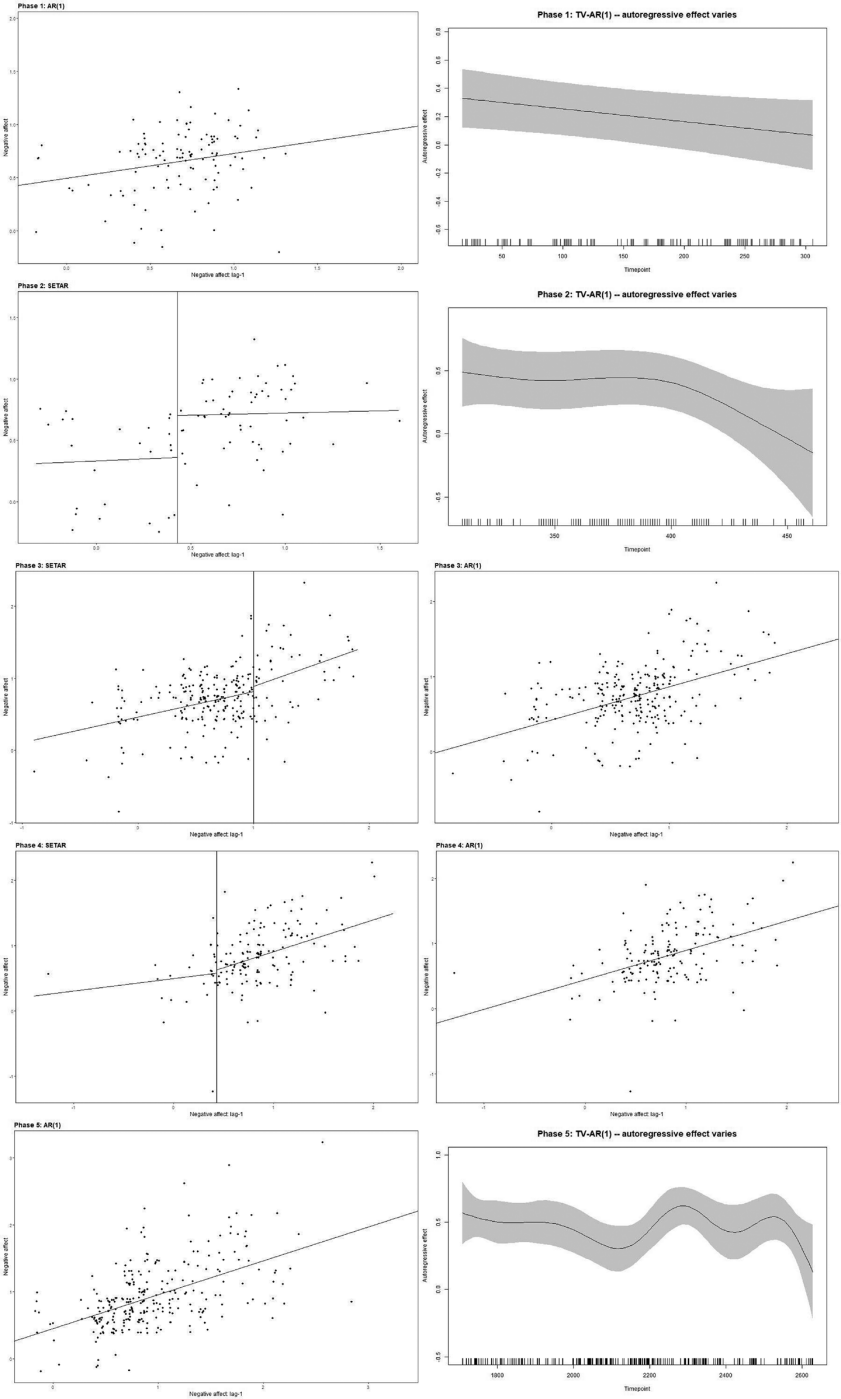
Selected models for describing negative affect dynamics across phases.

In this section, we re‐analyse a 239‐day experience sampling dataset collected from one participant diagnosed with major depressive disorder (Kossakowski et al., 2017; Wichers et al., 2016), focusing on the 10‐time‐per‐day momentary measures of positive and negative affect.^9^ The 239 days can be viewed as five phases around a double‐blind (i.e. unknown to the participant and researchers) period of gradual discontinuation of the participant's antidepressant (see Figures 2 and 3 for details). The five phases include: (I) baseline (day 1–28); (II) double‐blind period without antidepressant reduction (day 29–42); (III) double‐blind period with antidepressant reduction (day 43–98); (IV) planned post‐assessment after antidepressant reduction (day 99–155); (V) additional post‐assessment (day 156–239). The patient presented a sudden increase in depressive symptoms on day 127 and soon resumed the use of antidepressants. Explicit changes in both the treatment and the participant's depressive symptoms led to the use of different nonstationary time‐series models for understanding the changing temporal dynamics (e.g., Albers & Bringmann, 2020; Cabrieto et al., 2018; Haslbeck et al., 2021; Wichers et al., 2016). These applications presented promising new models for nonstationarity but lacked a comprehensive comparison of these new models' explanatory and predictive values relative to benchmark models. In this re‐analysis, we fit the nine models used in the earlier simulation study to the data and conduct model selection with two purposes. First, we wish to directly investigate whether the previously proposed models are indeed optimal when more candidate models are considered. Second, given the findings of simulation conditions with non‐normal errors, we demonstrate a few perspectives on how to critically evaluate the model selection results based on imperfect empirical data (e.g. having missing values, non‐i.i.d. errors).

### 5.1 Descriptive statistics

Figure 3 and Table 7 suggest that the mean of positive affect does not differ across phases, whereas that of negative affect seems to differ. Because we fit AR(1)‐based models and do not impute missing values, each observation requires a valid lag‐1 value to be predictable. This leads to a difference between the responded and predictable sample sizes as shown in the table.

**TABLE 7:** Descriptive statistics of positive and negative affect by phase.

Phase	T: responded	T: predictable	PA: mean (SD)	NA: mean (SD)
1	176	110	4.06 (.69)	.65 (.32)
2	110	82	4.16 (.75)	.60 (.38)
3	385	248	4.08 (.75)	.70 (.41)
4	317	162	4.03 (.74)	.81 (.43)
5	485	271	4.04 (.77)	.90 (.49)
All	1473	873	4.06 (.74)	.78 (.44)

Abbreviations: All, the complete time‐series over the 239 days; NA, negative affect; PA, positive affect.

### 5.2 Results: model estimation and selection

We estimated each candidate model without imputing missing data to stay consistent with previous analyses of this dataset. The first observation of each day is not accompanied by a same‐day lagged value and thus not predicted by the previous observation during model estimations.^10^ Then, we calculated the ICs for the estimates of each candidate model and conducted LOOCV and 10‐block CV for all models except for the HMM and the RS‐AR(1) model. This is because the dynrR package (Ou et al., 2019) used for estimating both models cannot facilitate CV without imputing missing values. R scripts for reproducing all analyses conducted can be found at https://osf.io/ahj3u.

Tables 8 and 9 present the top two models selected by the ICs and CV for describing the dynamics of positive and negative affect. We visualize the top two models selected for describing the negative affect dynamics across five phases in Figure 3.

**TABLE 8:** Positive affect dynamics: results of model selection.

Phase	Rank	AIC	AICc	HQ	BIC	LOOCV	Blocked CV
1	1	TV‐AR(1): α varies	AR(1) with trend	AR(1) with trend	AR(1) with trend	AR(1) with trend	AR(1) with trend
	2	AR(1) with trend	TV‐AR(1): α varies	TV‐AR(1): α varies	TV‐AR(1): α varies	TV‐AR(1): α varies	TV‐AR(1): α varies
2	1	TV‐AR(1): ϕ varies	TV‐AR(1): ϕ varies	SETAR	SETAR	TV‐AR(1): ϕ varies	AR(1)
	2	SETAR	AR(1)	AR(1)	AR(1)	AR(1)	AR(1) with trend
3	1	AR(1)	AR(1)	AR(1)	AR(1)	AR(1)	AR(1)
	2	SETAR	SETAR	SETAR	TV‐AR(1): ϕ varies	AR(1) with trend	AR(1) with trend
4	1	SETAR	SETAR	SETAR	SETAR	AR(1)	AR(1)
	2	TV‐AR(1): α varies	TV‐AR(1): α varies	AR(1)	AR(1)	AR(1) with trend	AR(1) with trend
5	1	SETAR	SETAR	SETAR	SETAR	AR(1)	AR(1)
	2	AR(1)	AR(1)	AR(1)	AR(1)	AR(1) with trend	AR(1) with trend
All	1	SETAR	SETAR	SETAR	SETAR	AR(1)	AR(1)
	2	AR(1)	AR(1)	AR(1)	AR(1)	TV‐AR(1): ϕ varies	AR(1) with trend

**TABLE 9:** Negative affect dynamics: results of model selection.

Phase	Rank	AIC	AICc	HQ	BIC	LOOCV	Blocked CV
1	1	RS‐AR(1)	RS‐AR(1)	SETAR	AR(1)	TV‐AR(1): ϕ varies	TV‐AR(1): ϕ varies
	2	TV‐AR(1): ϕ varies	TV‐AR(1): ϕ varies	TV‐AR(1): ϕ varies	SETAR	AR(1) with trend	AR(1)
2	1	RS‐AR(1)	RS‐AR(1)	RS‐AR(1)	SETAR	TV‐AR(1): α varies	TV‐AR(1): ϕ varies
	2	TV‐AR(1): α varies	HMM	SETAR	RS‐AR(1)	TV‐AR(1): ϕ varies	AR(1)
3	1	RS‐AR(1)	RS‐AR(1)	RS‐AR(1)	SETAR	AR(1)	AR(1)
	2	SETAR	SETAR	SETAR	RS‐AR(1)	AR(1) with trend	AR(1) with trend
4	1	TV‐AR(1): ϕ varies	TV‐AR(1): ϕ varies	SETAR	SETAR	AR(1)	AR(1)
	2	SETAR	SETAR	AR(1)	AR(1)	AR(1) with trend	AR(1) with trend
5	1	RS‐AR(1)	RS‐AR(1)	RS‐AR(1)	RS‐AR(1)	TV‐AR(1): α varies	AR(1)
	2	SETAR	SETAR	SETAR	SETAR	AR(1)	TV‐AR(1): α varies
All	1	RS‐AR(1)	RS‐AR(1)	RS‐AR(1)	SETAR	AR(1) with trend	AR(1) with trend
	2	SETAR	SETAR	SETAR	RS‐AR(1)	TV‐AR(1): α varies	TV‐AR(1): ϕ varies

The AR(1) models (with or without a trend) are often selected by ICs and CV to describe the dynamics of positive affect, with the SETAR model being another common preference. Thus, models assuming no change or abrupt changes are preferred, which conflicts with the gradually increasing autocorrelation found for the detrended time‐series by Albers and Bringmann (2020).

For negative affect, models involving multiple regimes (i.e. SETAR and RS‐AR(1)) are often selected by the ICs while the two CV approaches prefer other candidate models without regime‐switching. When the models with multiple regimes are selected, checking if parameter estimates are of reasonable values (e.g. whether the autocorrelation estimate in each regime is in the range of (0, 1)) can further help avoid incorrectly selecting an overspecified model. For the SETAR models estimated for the temporal dynamics of negative affect,^11^ this criterion is only met in phases 2, 3 and 4, whereas for the RS‐AR(1) models, this criterion is only met in phases 2 and 4. Therefore, although BIC performs well in the simulation study, researchers should not blindly rely on it for model selection with empirical data. Our findings based on the selected models are generally consistent with previous studies, except that we selected the stationary AR(1) model for phase 4, which did not support the critical slowing down (i.e. increased autocorrelation) discovered in previous studies (Albers & Bringmann, 2020; Cabrieto et al., 2018; Wichers et al., 2016). The differences are again partly due to the fact that we did not detrend the time series as in the previous studies. Such findings show that small differences in the methodological choices can lead to very different conclusions (Bastiaansen et al., 2020) and that the robustness of critical slowing down should be further investigated (Helmich et al., 2024).

## 6 DISCUSSION

In this paper, we evaluated ICs and prediction‐based model selection techniques (cross‐validation and out‐of‐sample prediction) in their ability to identify AR(1)‐based nonstationary processes. In the simulation study, OOS prediction often had optimal performance under adequate sample size (T≥100). This is not surprising as our simulation setting only concerns the parametric framework, where the true model is one of the candidate models. When the true model can be estimated accurately, OOS prediction naturally demonstrates the best predictive accuracy. BIC performed well in most simulation conditions under adequate sample size, showing its ability to balance the risk of selecting under‐specified and overspecified models. Other ICs and CV all suffered from certain problems such as selecting overspecified models (AICc under large sample sizes, HQ under small sample sizes and AIC and LOOCV consistently) and over‐extrapolation (e.g. blocked CV performs unsatisfactorily when the time‐varying pattern is different between the training and validation set, such as for a TV‐AR(1) model). In general, identifying nonstationarity patterns caused by changes in autocorrelation was more challenging than identifying those caused by changes in the AR(1) model's intercept.

From the re‐analysis of empirical data, we discovered the different preferences by the ICs (for multiple‐regime models) and CV (for models without regime‐switching). The common preference of ICs of the SETAR model warrants rethinking whether ICs that do not penalize the threshold are appropriate when comparing the threshold model with other models (Hamaker et al., 2009). Additionally, we argue that data‐driven model selection should be reconsidered if the estimates from multiple‐regime models are inconsistent with theoretical expectations. Identifying such implausible estimates is crucial for preventing faulty inferences of the temporal dynamics.

Our simulation study also comes with limitations. First, the simulation study we conducted relies on the assumption that the true data generating model can be identified and is among the candidate models we fit to the data (sometimes referred to as the M‐closed setting, Bernardo & Smith, 2000). This assumption can become unrealistic with empirical data analysis, where the true data generating model is usually not among the candidate models, or where the true data generation mechanism cannot even be specified in a model. In such situations, the suggestions we made based on the current simulation study can thus be of limited value. In these cases, we therefore recommend researchers to complement model selection approaches with the incorporation of theoretical knowledge about the underlying process. Second, we rely on frequentist ICs and predictive accuracy calculated from CV and OOS prediction for model selection. Both ICs and CV come with assumptions that are not necessarily met with empirical time‐series data: normal errors for ICs and independent training and test data for CV. Future work on adapting both approaches to fit the characteristics of psychological time‐series data is imperative (e.g., independent validation; leave‐future‐out CV; Bürkner et al., 2020; Braun et al., 2023). Moreover, all approaches we used are purely data‐driven and do not exhaust the available model selection techniques. Future studies can consider investigating the performance of other model selection techniques in similar simulation settings, for instance, Bayesian model averaging (Claeskens & Hjort, 2008) and theory‐based model selection (Kuiper, 2022). Third, although we simulated data based on nine different candidate models with 31 different settings, the simulation conditions might still not be extensive enough to cover all possible nonstationary patterns. For example, simulation conditions for the HMM and RS‐AR(1) model only allow regime‐switching at fixed time‐points (i.e. $\frac{T}{4},\frac{T}{2},\frac{3T}{4}$, similar to the settings used in Cabrieto et al. (2018)), which results in very low probability of regime‐switching. However, empirical studies usually find frequent switches between regimes (e.g., Hamaker & Grasman, 2012; Stifter & Rovine, 2015), suggesting that our simulation conditions selected for these two models might not be highly representative of empirical data. Hence, it is worth investigating whether the assessed model selection techniques perform differently when regime switches are more frequent.

Another reflection is that missing data, which is inevitable in ESM studies, poses challenges at multiple steps in the time‐series analysis workflow. The statistical tests of unit‐root and deterministic trends typically require a time‐series without missing observations (Dickey & Fuller, 1979; Fowler et al., 2024; Kwiatkowski et al., 1992; Phillips & Perron, 1988; Ryan et al., 2023) and thus could not be applied in our empirical analysis. Estimation of the HMM and RS‐AR(1) model through dynr also recommends imputing missing data and fails when any external covariate contains missing values (Ou et al., 2019). Although researchers have developed imputation techniques for intensive longitudinal data (Buuren & Groothuis‐Oudshoorn, 2011; Ji et al., 2018; Li et al., 2019; Liu & Molenaar, 2014), such techniques usually assume stationarity or a time‐invariant missing data mechanism, which are unrealistic for nonstationary time‐series. Future research should first actively investigate the missing data mechanism in nonstationary time‐series. Based on the findings, researchers can further evaluate whether imputation is a feasible approach under nonstationarity.

This paper focuses on AR(1)‐based idiographic univariate time‐series analysis. While this approach is valuable, it is essential to recognize that researchers often conduct multiple‐subject, multivariate analyses of ESM data to examine average temporal dynamics among multiple variables across participants (Blanchard et al., 2023; Bringmann et al., 2013; de Vos et al., 2017; Ebrahimi et al., 2021; Epskamp et al., 2018; Fisher et al., 2022; Fried et al., 2022; Wigman et al., 2015). Furthermore, they can identify meaningful subgroups of participants with similar dynamics (Crawford et al., 2024; Ernst et al., 2023, 2020; Fisher et al., 2024). Typically, such analyses rely on the (multilevel) vector autoregressive (VAR) model to capture these complex relationships (Lütkepohl, 2005). Using multiple‐subject, multivariate models presents additional challenges, as assessing nonstationarity for multiple individuals and variables requires considerable effort to consider each participant and be able to model simultaneously the relations between variables. While assessing and modeling univariate nonstationarity is a crucial first step, future research should also focus on how to best assess nonstationarity in the multivariate and multi‐subject context.

This study provides the first systematic account of the performances of data‐driven model selection techniques when applied to simulated nonstationary time‐series. We recommend BIC, considering its simplicity and ability to balance under‐ and over‐fitting. When selecting the model with optimal predictive accuracy in nonstationary time‐series, blocked‐CV presents a similar tendency of underfitting, as shown by Liu and Zhou (2023) in model selection for stationary time‐series. Future research should thus investigate how to minimize the bias of CV in time‐series analysis (Bates et al., 2023; Cooper et al., 2023). Additionally, we show how researchers can complement these techniques with their knowledge when analysing empirical data. Model building and selection for nonstationarity remain challenging tasks, as slight methodological differences can yield vastly different results. Thus, we recommend that future research balances data‐ and theory‐driven approaches in nonstationary time‐series analysis.

## AUTHOR CONTRIBUTIONS

**Yong Zhang:** conceptualization; investigation; funding acquisition; writing – original draft; methodology; validation; visualization; writing – review and editing; software; formal analysis; project administration; data curation; resources. **Anja F. Ernst:** conceptualization; investigation; funding acquisition; methodology; writing – review and editing; software; supervision. **Ginette Lafit:** conceptualization; investigation; funding acquisition; writing – review and editing; methodology; software; supervision. **Ward B. Eiling:** investigation; writing – original draft; methodology; validation; visualization; software; formal analysis; data curation. **Laura F. Bringmann:** conceptualization; investigation; funding acquisition; writing – review and editing; methodology; supervision.

## DISCLOSURE OF ARTIFICIAL INTELLIGENCE‐GENERATED CONTENT (AIGC) TOOLS

The authors used ChatGPT for improving spelling, grammar and general editing. The authors reviewed the content generated and takes full responsibility for the content of the article.

## CONFLICTS OF INTEREST

The authors declare that they have no known competing financial interests or personal relationships that could have appeared to influence the work reported in this paper.

## Supporting information


Appendix S1.

## Data Availability

The data that support the findings of this study are openly available in OSF storage at https://osf.io/ahj3u.

## Footnotes

^1^
A detailed protocol of the review can be found in the https://osf.io/ahj3u.

^2^
Specifically, nonstationarity was neither tested nor accounted for in their modeling approach.

^3^
It is worth noting that Belkin et al. (2019) shows that machine learning models such as neural networks do not comply with this trade‐off. They are overparameterized but can achieve optimal predictive accuracy for unseen data. Such models are beyond the scope of this paper.

^4^
Asymptotically refers to what happens as the sample size becomes extremely large.

^5^
This refers to non‐missing observations that also have non‐missing values for the predictor.

^6^
When log(log(T))=1, $T=e^{e^{1}}=15$, the HQ is equivalent to the AIC. However, to achieve a stronger penalty than the AIC, such as log(log(T))=2, $T=e^{e^{2}}=1618$.

^7^
When the data‐generating model is the stationary AR(1) model, the extent of nonstationarity is not considered.

^8^
A TV‐AR(1) model with time‐varying intercept is equivalent to the AR(1) model with a linear trend in the extreme case where edf=1.

^9^
We operationalize positive and negative affect as the unweighted mean of the corresponding items: positive affect – strong, relaxed, cheerful, satisfied and enthusiastic; negative affect – irritated, lonely, anxious, guilty, indecisive, down, suspicious. R scripts and complete results can be found in https://osf.io/ahj3u.

^10^
Interested readers can refer to Berkhout et al. (2025) for a more nuanced discussion of this methodological decision.

^11^
The detailed model estimates are available in online supplemental materials.
