# Supplementary material for: An investigation into in‐sample and out‐of‐sample model selection for nonstationary autoregressive models
Source: Br J Math Stat Psychol. 2025 Oct 28;79(2):409–36. doi: 10.1111/bmsp.70012 (PMC13067993; doi:10.1111/bmsp.70012)
Supplement: Supplementary file 1 — Appendix S1. [file BMSP-79-409-s001.pdf]

Appendix A

Details on the time-series models we study

In this section, we present more details on the univariate time-series models that we study.

AR(1) model

As the basis of all models discussed in this study, the AR(1) model can be written as:

$$y_t = \alpha + \phi y_{t-1} + \epsilon_t, \tag{A1}$$

with α being the intercept, ϕ the autoregressive effect, and ϵ_t the independent and identically normally distributed innovation term which represents random fluctuations in the time-series:

$$\epsilon_t \overset{\text{iid}}{\sim} \mathcal{N}(0, \sigma^2). \tag{A2}$$

An AR(1) model can generate stationary time-series if $|\phi| < 1$ (Hamilton, 1994). The mean and variance of an AR(1) process can be calculated as:

$$E(y_t) = \frac{\alpha}{1-\phi}, \tag{A3}$$

$$var(y_t) = \frac{\sigma^2}{1-\phi^2}, \tag{A4}$$

which will be helpful for further understanding the nonstationarity of other models we introduce.

To get an intuition of how the autocorrelation influences the temporal dynamics, here, we simulated and visualized time-series following three AR(1) models: $\phi = 0, .3, .7$, respectively (Figure A1). An AR(1) model with $\phi = 0$ is also called a *white noise* model since there is no temporal dependency in the generated time-series. From the plots, we notice that when the innovation variance (σ^2) is fixed, larger autocorrelation (ϕ) leads to smoother time-series: the current value is more likely to resemble the previous value and less influenced by random fluctuations. Yet, the difference in autocorrelation is still

difficult to spot with only visual inspection. For this reason, researchers can further use the partial autocorrelation function to get a more accurate view of the temporal dependency in the time-series.

Figure A1

AR(1) models with different autocorrelations

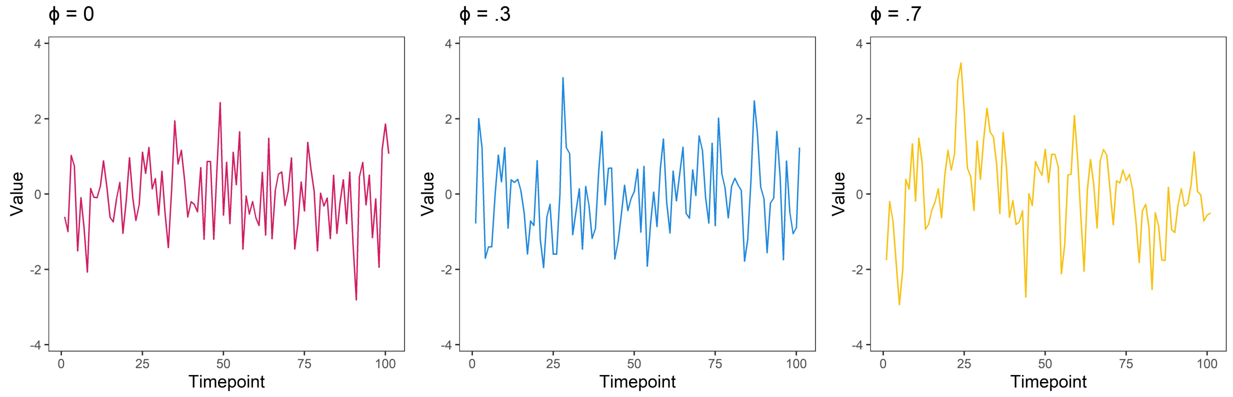

Note. For all three models, $\alpha = 0, \sigma = 1$.

Random walk

A random walk model is expressed as follows:

$$y_t = \alpha + y_{t-1} + \epsilon_t, \tag{A5}$$

with $\epsilon_t \sim \mathcal{N}(0, \sigma^2)$. Random walk is thus a special case of the AR(1) model where $\phi = 1$: an observation "walks" from the current time-point (y_t) to the next time-point (y_{t+1}) in a "random" manner (i.e., added by a fixed trend term, α, and a random term, ϵ_t). For simplicity, we only consider the random walk models without the trend term (i.e., $\alpha = 0$).

Given that $|\phi| < 1$ does not hold for a random walk, the time-series generated with this model is nonstationary. More precisely, the mean and variance of a random walk process at a time-point t can be calculated as:

$$\begin{aligned} y_t &= \alpha + y_{t-1} + \epsilon_t \\ &= \alpha + (\alpha + y_{t-2} + \epsilon_{t-1}) + \epsilon_t \\ &= 2\alpha + (\alpha + y_{t-3} + \epsilon_{t-2}) + (\epsilon_t + \epsilon_{t-1}) \\ &= \cdots \\ &= t\alpha + y_0 + \sum_{i=1}^{t} \epsilon_i \end{aligned} \tag{A6}$$

$$\begin{aligned} E(y_t) &= E(t\alpha + y_0 + \sum_{i=1}^{t} \epsilon_i) \\ &= t\alpha + y_0 \end{aligned} \tag{A7}$$

$$\begin{aligned} var(y_t) &= var(t\alpha + y_0 + \sum_{i=1}^{t} \epsilon_i) \\ &= t\sigma^2. \end{aligned} \tag{A8}$$

We can observe that the mean and variance of a random walk is a function of t in Figure A2, where four time-series following the random walk process with $\sigma^2 = 1$ are visualized. The variance of the time-series (i.e., the dispersion of different time-series at the same time-point) increases with time whereas the mean (the center of different time-series at the same time-point) stays constant since $\alpha = 0$.

Figure A2

Four time-series following the same random walk process

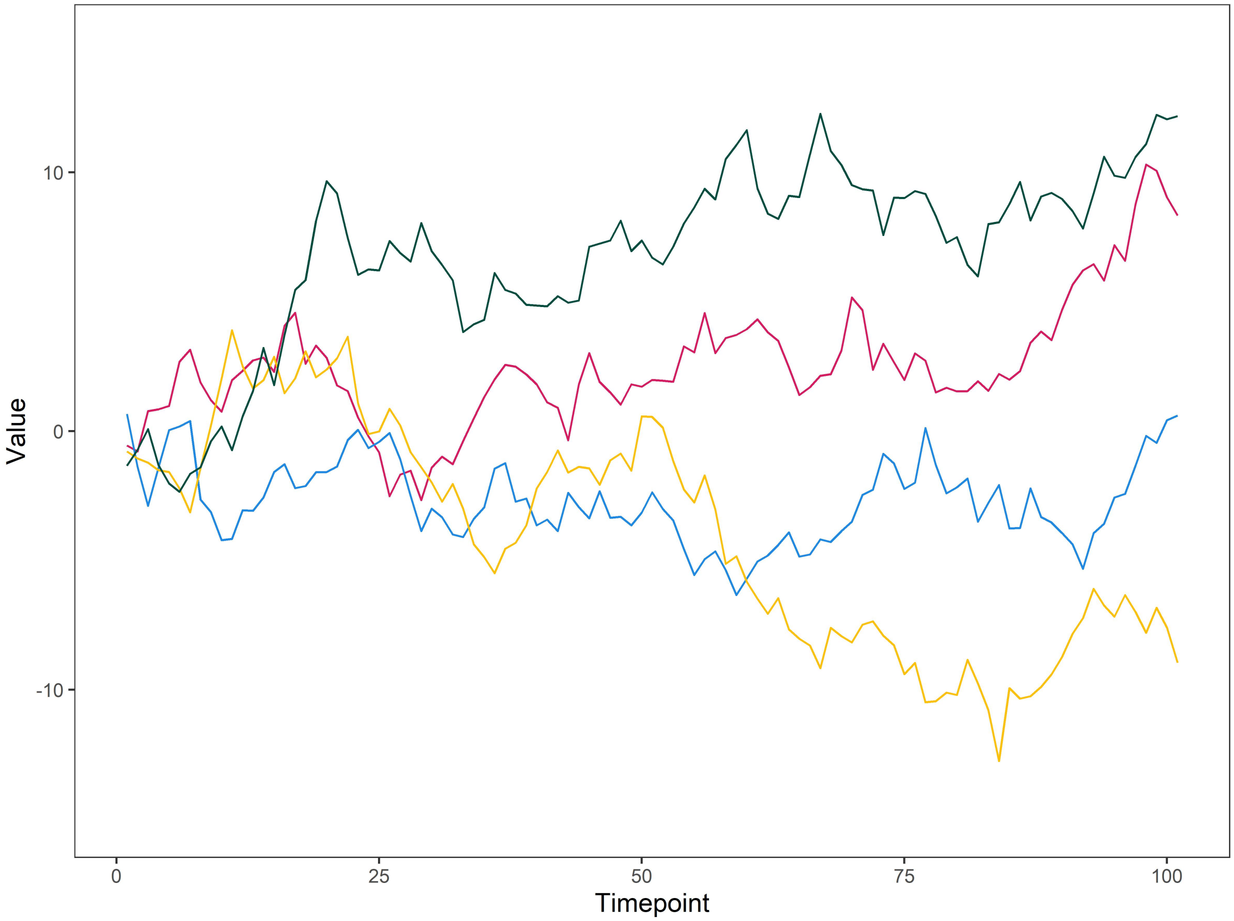

AR(1) model with deterministic trends

An AR(1) model with a linear trend is expressed as:

$$y_t = \alpha + \beta t + \phi y_{t-1} + \epsilon_t, \tag{A9}$$

with β representing the linear time trend and $\epsilon_t \sim \mathcal{N}(0, \sigma^2)$. This model also leads to nonstationary time-series whose mean varies over time due to the trend whereas the variance is constant.

In Figure A3, two time-series generated from this model are visualized: $\alpha = 0, \phi = .3$, and $\sigma = 1$ for both time-series, yet $\beta = .1$ and $.5$ respectively. The change of mean is noticeably more drastic for the time-series with a stronger trend $\beta = .5$.

Figure A3

Two time-series generated by AR(1) models with different trends

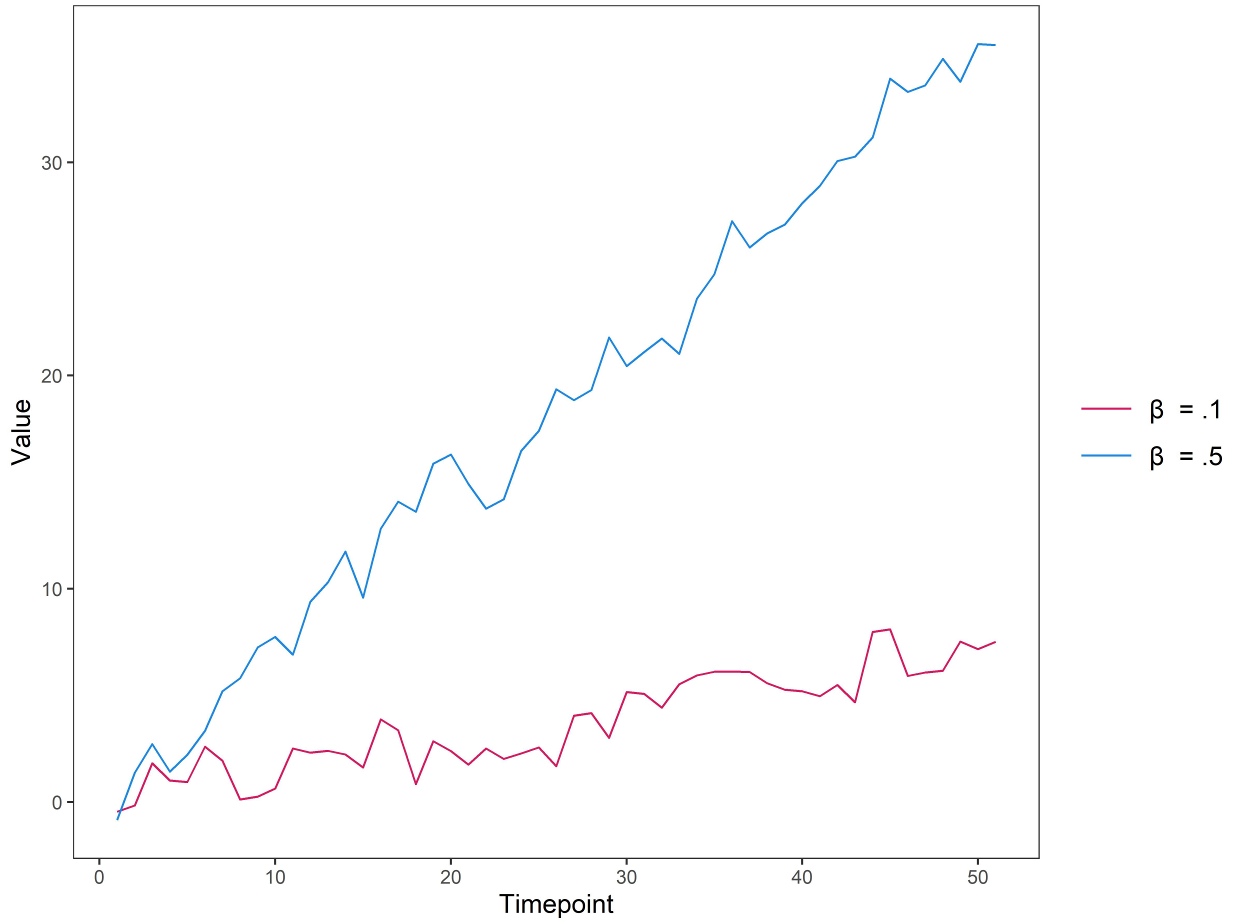

Time-varying AR(1) model

The *time-varying AR(1) model* ((TV-AR(1)); Bringmann et al., 2017) is a variation of the stationary AR(1) model, where the model parameters are allowed to vary over time. Such a model can be written as follows:

$$y_t = \alpha_t + \phi_t y_{t-1} + \epsilon_t, \tag{A10}$$

with $\epsilon_t \sim \mathcal{N}(0, \sigma^2)$. This semiparametric model depicts the time-varying parameters as smooth functions of time, allowing them to vary over time gradually. Therefore, abrupt

changes cannot be accurately estimated by this model without additional specification. Moreover, the TV-AR(1) model requires that $|\phi_t| < 1$ across the time-series, which ensures local stationarity (Dahlhaus, 1997), suggesting that the mean and variance at a given time-point t can be calculated as:

$$E(y_t) \approx \frac{\alpha_t}{1-\phi_t}, \tag{A11}$$

$$var(y_t) \approx \frac{\sigma^2}{1-\phi_t^2}. \tag{A12}$$

As an example, we consider two models, each with one parameters varying over time in a sine-wave. Model 1 involves intercept changes: $\alpha_t = \sin\frac{2\pi t}{50}$, $\phi = .3$, and $\sigma = 1$. Whereas in model 2, the autocorrelation varies over time: $\alpha = 0$, $\phi_t = .3 + 0.1 \sin\frac{2\pi t}{50}$, and $\sigma = 1$. The two time-series and corresponding changing parameters are visualized in Figure A4. These two plots show that the change of autocorrelation is harder to detect through visual inspection than of the mean caused by the change of intercept.

Figure A4

Two time-series generated by the TV-AR(1) models with changes in either intercept or autocorrelation

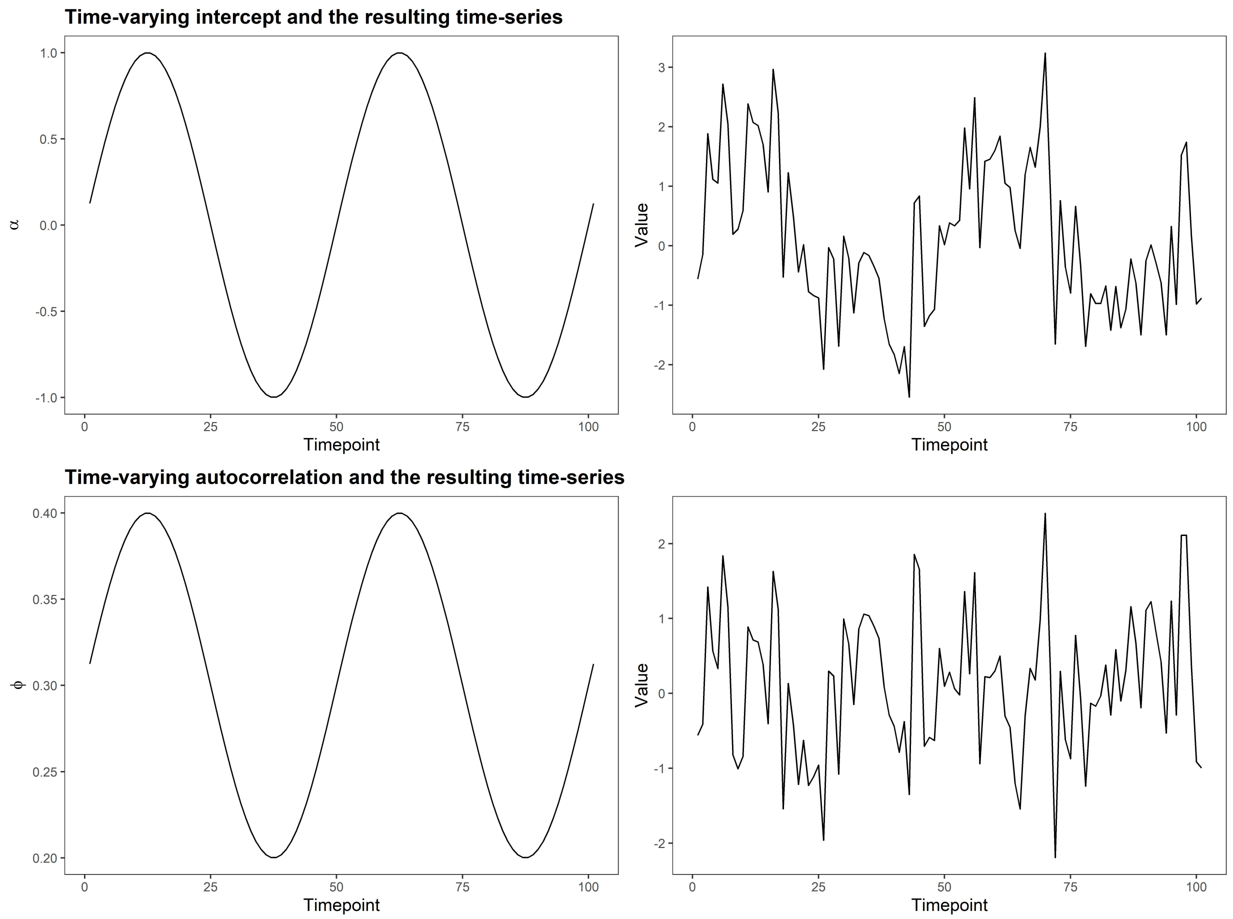

Threshold autoregressive model

The threshold autoregressive model characterizes processes that have multiple autoregression-based regimes and switch between regimes whenever the value of a threshold variable crosses the threshold. This threshold variable can be either endogenous (e.g., the lagged outcome variable, y_{t-1}) or exogenous (e.g., a contextual variable, z_t). As a simplest example, Hamaker et al. (2009) studied the following process:

$$y_t = \begin{cases} \alpha^{(1)} + \phi^{(1)} y_{t-1} + \epsilon_t^{(1)}, & \text{if } y_{t-1} \leq \tau \\ \alpha^{(2)} + \phi^{(2)} y_{t-1} + \epsilon_t^{(2)}, & \text{if } y_{t-1} > \tau \end{cases} \tag{A13}$$

with $\epsilon_t^{(1)} \sim \mathcal{N}(0, \sigma^{(1)2})$ and $\epsilon_t^{(2)} \sim \mathcal{N}(0, \sigma^{(2)2})$. This model has two regimes, each is an AR(1) model itself. Besides, the threshold variable that triggers regime switching is the endogenous lagged outcome variable, y_{t-1}. Therefore, this model is called a *self-exciting threshold autoregressive model*, or SETAR(2,1,1).

Again, we simulate and visualize two time-series following the SETAR(2,1,1) models in Figure A5. The simulation setting is the same as in Hamaker et al. (2009). The two figures (the time-series plot of y_t against time-point, and the state-space plot of y_t against y_{t-1}) on the first row are for the time-series generated with a SETAR(2,1,1) model with different intercepts across the two regimes. Whereas the figures on the bottom row show the time-series generated with a SETAR(2,1,1) model with different autoregressive coefficients in the two regimes. As shown in equations A3 and A4, the mean and variance of an AR(1) process is related to α and ϕ, and ϕ and σ, respectively. As such parameters can differ across regimes in the SETAR(2,1,1) model, such model will also generate nonstationary time-series. However, it is difficult to tell from the time-series plots whether and which specific type of nonstationarity exists, demonstrating the importance of proper model selection techniques in this task.

Figure A5

Two time-series generated by SETAR(2,1,1) models with different parameters

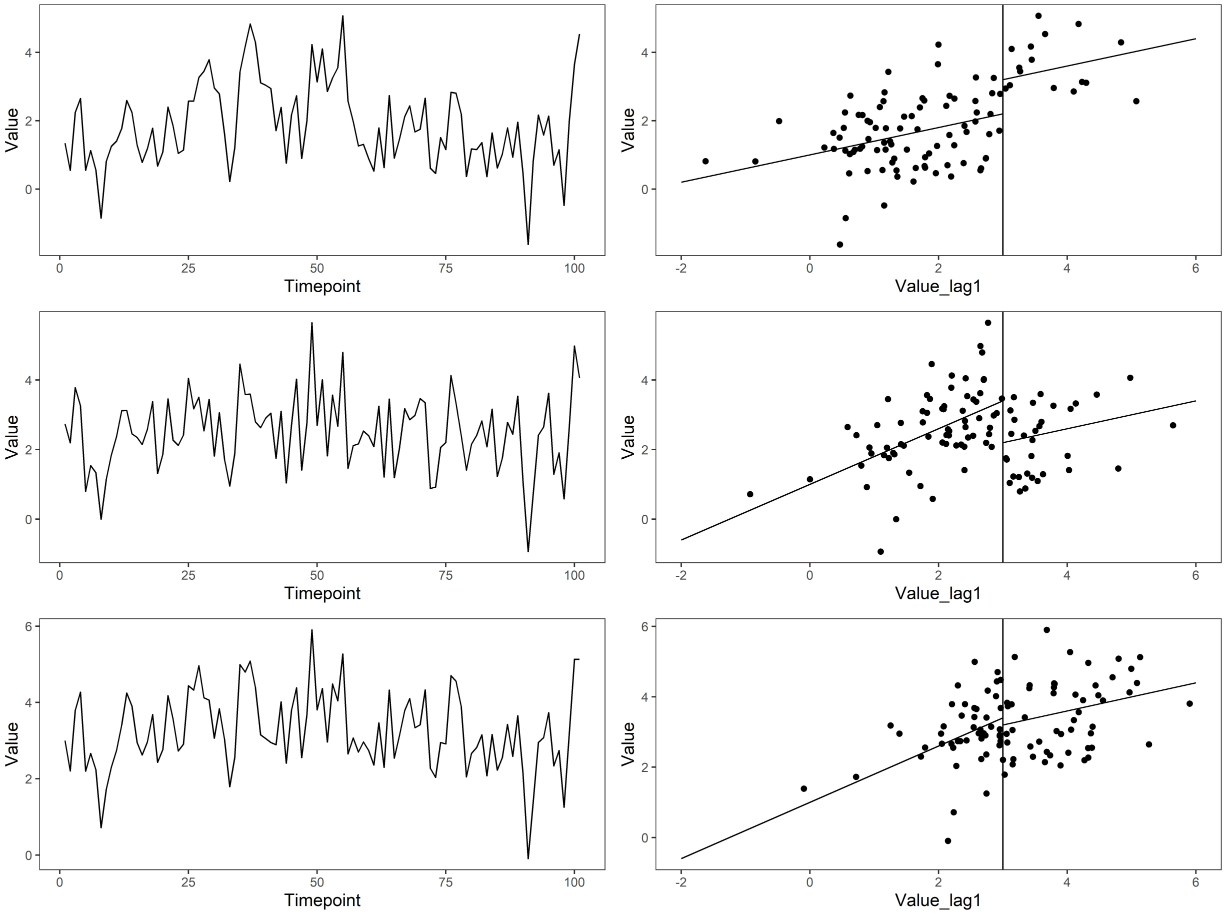

Note. For both time-series, $\sigma^{(1)2} = \sigma^{(2)2=1}$, $\tau = 3$. Specifically for the top time-series, $\alpha^{(1)} = 1, \alpha^{(2)} = 2, \phi^{(1)} = \phi^{(2)} = .4$, and for the bottom time-series, $\alpha^{(1)} = \alpha^{(2)} = 1, \phi^{(1)} = .8, \phi^{(2)} = .4$.

Hidden Markov model

The hidden Markov model (HMM) is another type of regime-switching model that allows the switching to happen in a probabilistic manner (Hamaker & Grasman, 2012; Zucchini & MacDonald, 2009). A simple two-regime HMM can be specified as follows:

$$y_t = \begin{cases} \alpha^{(1)} + \epsilon_t^{(1)}, & \text{if } s_t = 1 \\ \alpha^{(2)} + \epsilon_t^{(2)}, & \text{if } s_t = 2 \end{cases} \tag{A14}$$

with the innovation term in both regimes being normally distributed: $\epsilon_t^{(1)} \sim \mathcal{N}(0, \sigma^{(1)2}), \epsilon_t^{(2)} \sim \mathcal{N}(0, \sigma^{(2)2})$. s_t denotes the current regime at time-point t. The

temporal dependency is not modeled as autoregressive effects in HMM, and is instead reflected in the regime-switching. The probability of the current regime depends on the previous regime and is represented by the following transition matrix:

$$\Gamma = \begin{array}{r} \\ s_{t-1}=1 \\ s_{t-1}=2 \end{array} \begin{array}{c} \begin{array}{cc} s_t=1 & s_t=2 \end{array} \\ \begin{pmatrix} p_{11} & p_{12} \\ p_{21} & p_{22} \end{pmatrix} \end{array},$$

For example, p_{12} is the probability of switching from regime 1 to regime 2 at the next time-point: $p(s_t = 2|s_{t-1} = 1)$.

Time-series generated by this model can thus be seen as switching between two white-noise processes with different means. Here, we visualize a time-series following a HMM. The time-series contains 101 time-points and only switches between the two regimes twice: when $t = 51$ and $t = 101$ (Figure A6). The difference between intercepts of the two regimes is easily noticeable in this case due to the large extent of the difference and high probability of staying in the same regime. When there are smaller differences and more switches between regimes, visual inspection of the time-series will again not be enough to identify the actual process.

Figure A6

Time-series generated by a two-regime HMM

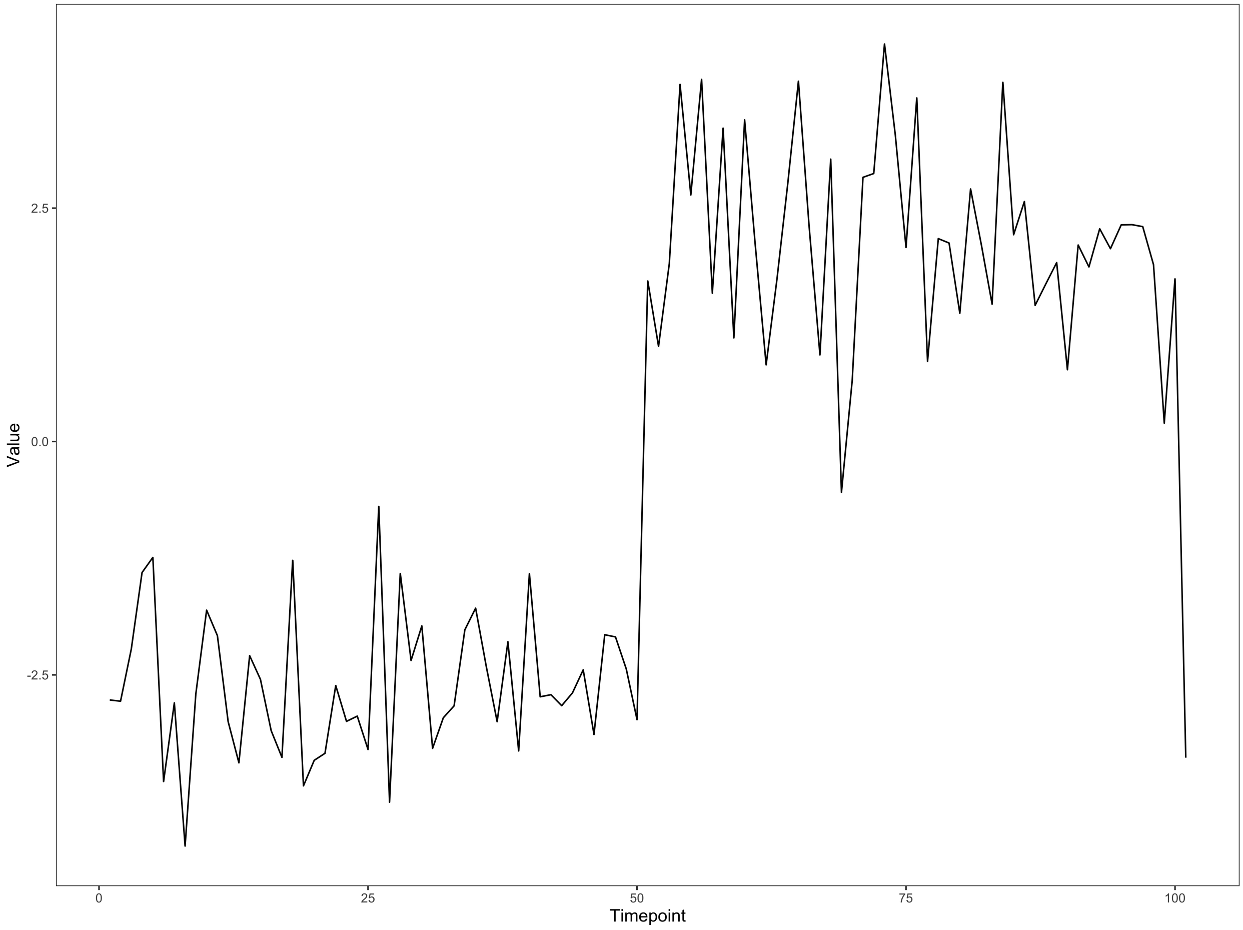

Note. The time-series is generated with a HMM with the following parameters: $\alpha^{(1)} = -2.5$, $\alpha^{(2)} = 2.5$, $\sigma^{(1)} = \sigma^{(2)} = 1$, $p_{11} = p_{22} = .98$, $p_{12} = p_{21} = .02$.

Regime-switching AR(1) model

By combining an AR(1) model with HMM, we get a regime-switching AR(1) (RS-AR(1)) model (Hamaker & Grasman, 2012), which generates time-series that switches between different AR(1) processes. A two-regime RS-AR(1) model can thus be written as:

$$y_t = \begin{cases} \alpha^{(1)} + \phi^{(1)} y_{t-1} + \epsilon_t^{(1)}, & \text{if } s_t = 1 \\ \alpha^{(2)} + \phi^{(2)} y_{t-1} + \epsilon_t^{(2)}, & \text{if } s_t = 2 \end{cases} \tag{A15}$$

with the innovation variance and transition matrix identical to those specified in HMM. Thus, with the RS-AR(1) model, the temporal dependency is resulted by both the

autocorrelation and the regime-switching.

Figure A7 presents a time-series generated by the following RS-AR(1) model used in Cabrieto et al. (2018). The time-series begins in regime 1 and switches once at $t = 51$.

Figure A7

Time-series generated by a two-regime RS-AR(1) model

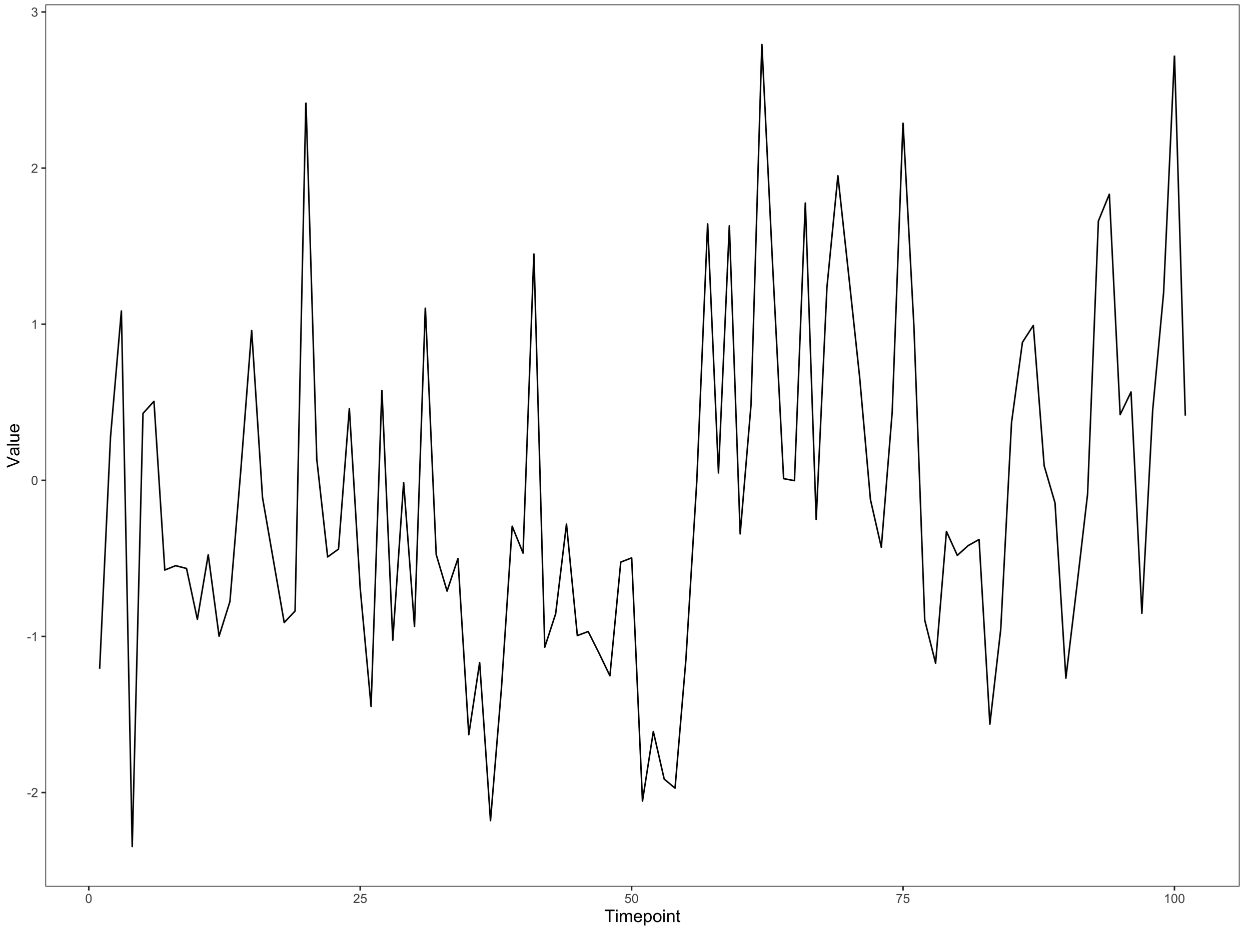

Note. The time-series is simulated from a RS-AR(1) model with the following parameters:

$$\alpha^{(1)} = \alpha^{(2)} = 0,\ \phi^{(1)} = 0,\ \phi^{(2)} = .5,\ \sigma^{(1)} = \sigma^{(2)} = 1$$

Appendix B

More details on K-L discrepancy

With continuous outcome variables, K-L discrepancy assesses the similarity between the true probability density (likelihood) function, $g(x)$, and the likelihood function given a candidate model, $f(x)$ (Konishi & Kitagawa, 2008):

$$\begin{aligned} \text{K-L} &= E_G\left[log\left\{\frac{g(x)}{f(x)}\right\}\right] \\ &= E_G[log(g(X))] - E_G[log(f(X))], \end{aligned} \tag{B1}$$

where E_G denotes the expectation with respect to the true distribution G. Smaller discrepancy represents higher similarity between $F(x)$ and $G(x)$, thus higher similarity between the candidate model and the true model.

Using this function, the goal of model selection becomes finding the model with the largest expected log-likelihood ($E_G[log(F(X))]$) among all candidate models. However, the log-likelihood of a statistical model estimated by the maximum likelihood method is a biased estimator of the expected log-likelihood, and this bias increases with model complexity (Konishi & Kitagawa, 2008). For this reason, log-likelihood-based model selection tools such as AIC were developed as an intended unbiased estimator of the K-L discrepancy by correcting this bias.

Appendix C

Model estimation and selection

Model estimation

We summarize the estimation method for each candidate model used in the current simulation study and provide example R code (R Core Team, 2021). All R code used in this study can be found in online supplemental materials (https://osf.io/ahj3u/?view_only=8e946b6507d14ed78d0b1e6130d5db1d).

We use the ordinary least squares (OLS) estimator to estimate the following models: AR(1), white noise, random walk, AR(1) with linear trend, and SETAR(2,1,1). The TV-AR(1) model is estimated with the generalized additive model (GAM; Hastie & Tibshirani, 1999; Wood et al., 2016), which applies smooth functions to the covariates and sums these functions. A TV-AR(1) model estimated with GAM can be written as:

$$\begin{aligned} y_t &= \alpha_t + \phi_t y_{t-1} + e_t \\ &= f_1(t) \cdot 1 + f_2(t) \cdot y_{t-1} + e_t, \end{aligned} \tag{C1}$$

where f_1 and f_2 are smooth functions for estimating the time-varying intercept and autocorrelation, respectively. In this study, we estimate two such models for each time-series: either intercept or autocorrelation varies. Each smooth function is estimated using thin plate regression splines with 10 basis functions as in Bringmann et al. (2017).

We estimate the SETAR(2,1,1) model in the same way as Berkhout et al. (2023) and Hamaker et al. (2009). First, all values of y_{t-1} are sorted in an ascending order to create a new set $\{\tilde{y_{t-1}}\}$. A key step is to choose the threshold value τ that distinguishes the two regimes from this set. As we want to ensure each regime contains at least 5% of all observations, we delete the smallest 5% and largest 5% from the set. We then start the iterative process of using one value from this trimmed set (y_i) as the potential threshold value and estimating two separate AR(1) models for observations with $y_{t-1} \leq y_i$ and $y_{t-1} > y_i$, until we have used all values as the threshold value. In the resulting

SETAR(2,1,1) models, the one that leads to the minimal residual sum of squares (RSS) are used as the model estimates.

The two models based on Markov Chains, the HMM and the RS-AR(1) model, are estimated with the state-space modeling framework. A log-likelihood function is constructed through a Kalman Filter, and resulting parameters are estimated via maximum likelihood. To minimize model complexity, we only estimate 2-regime models and constrain the innovation variances to be equal ($\sigma^{(1)2} = \sigma^{(2)2}$) when estimating both models.

Table C1 summarizes the estimation method and provides example R code for estimating each candidate model.

Table C1

Estimation methods and R code for all candidate models

Model	Estimation method	R function or package used
AR(1)	Ordinary least squares	`lm(value ~ value_lag1)`
White noise	Ordinary least squares	`lm(value ~ 1)`
Random walk	Ordinary least squares	`var(value - value_lag1)`
AR(1) with linear trend	Ordinary least squares	`lm(value ~ value_lag1 + time-point)`
TV-AR(1): intercept (α) change	GAM	`mgcv::gam(value ~ s(time-point, k = 10, bs = "tp") + value_lag1)`
TV-AR(1): autocorrelation (ϕ) change	GAM	`mgcv::gam(value ~ s(time-point, by = value_lag1, k = 10, bs = "tp") + value_lag1)`
SETAR(2,1,1)	Ordinary least squares	Same as in Berkhout et al. (2023)
HMM	Maximum likelihood	`dynr` (Ou et al., 2019)

RS-AR(1)	Maximum likelihood	`dynr` (Ou et al., 2019)

Note: The three variable names used in the code represent the following: "time-point" - the timestamp of the time-series ($t = 1, 2, 3, \cdots, T$); "value" - the value of the time-series at time-point t; "value_lag1" - the value of the time-series at time-point $t - 1$.

Model selection

In this section, we briefly discuss some additional points that clarify how the model selection techniques are applied to the candidate models.

For calculating the ICs, we require the following three pieces of information for each model: (1) the log-likelihood ($log(\mathcal{L})$), (2) the number of freely estimated parameters (p), and (3) the sample size (T). For models estimated through OLS, the log-likelihood was calculated based on the residual sum of squares (RSS; Soch et al., 2024):

$$log(\mathcal{L}) = -\frac{T}{2} log(RSS) - \frac{T}{2} - \frac{T}{2} log\left(\frac{2\pi}{T}\right). \tag{C2}$$

For most candidate models, determining p is straightforward (see Table C3). However, two candidate models require special attention when determining p. For TV-AR(1) models estimated with GAM, p consists of both the innovation variance (σ^2) and the parameters needed by the smooth functions to sufficiently describe the intercept and autocorrelation. This latter number is called the effective degrees of freedom (*edf*; Hastie & Tibshirani, 1999)[1]. Therefore, p can be calculated by $edf + 1$ for these models. For SETAR models, the threshold variable τ is not considered as a freely estimated parameter in the calculation of ICs (Wong & Li, 1998)[2].

[1] When estimating one smooth function, a result of $edf = 1$ indicates that the smooth function is simply a linear function of time.

[2] As argued by Hamaker et al. (2009), if the number of regimes is not fixed when estimating a SETAR model, it is important to use other IC that penalize the threshold parameter to avoid overfitting.

Table C3

Overview of freely estimated parameters in all candidate models

Model	Parameters to estimate	Number of freely estimated parameters (p)
AR(1)	α, ϕ, σ	3
White noise	α, σ	2
Random walk	σ	1
AR(1) with linear trend	α, β, ϕ, σ	4
TV-AR(1): α change	σ, smooth function of α	$edf + 1$
TV-AR(1): ϕ change	σ, smooth function of ϕ	$edf + 1$
SETAR(2,1,1)	$\alpha^{(1)}$, $\alpha^{(2)}$, $\phi^{(1)}$, $\phi^{(2)}$, $\sigma^{(1)}$, $\sigma^{(2)}$	6
HMM	$\alpha^{(1)}$, $\alpha^{(2)}$, σ, p_{11}, p_{22}	5
RS-AR(1)	$\alpha^{(1)}$, $\alpha^{(2)}$, $\phi^{(1)}$, $\phi^{(2)}$, σ, p_{11}, p_{22}	7

Note: Extensive descriptions of the model parameters can be found in Appendix A. *edf* represents the effective degrees of freedom for a GAM.

In CV and out-of-sample predictive accuracy analysis, a common key step in each iteration is predicting the test set using the acquired model estimates. For models that only use manifest (contrary to latent) variables as covariates or thresholds, prediction is straightforward. However, prediction with the two models that involve latent variables (i.e., the latent states), HMM and RS-AR(1), requires an additional step of estimating the state of each test observation (s_t). We specify the prediction rule of both models as a weighted average of the predicted values across all regimes (Hamilton, 1994):

$$\hat{y}_t = P(s_t = 1|y_{t-1})E[y_t|y_{t-1}, s_t = 1] + P(s_t = 2|y_{t-1})E[y_t|y_{t-1}, s_t = 2], \qquad \text{(C3)}$$

where s_t denotes the regime at time-point t. $P(s_t = 1|y_{t-1})$ and $P(s_t = 2|y_{t-1})$ are

estimated following a two-step procedure. First, we estimate $P(s_{t-1} = 1|y_{t-1})$ using the Bayes' rule:

$$P(s_{t-1} = 1|y_{t-1}) = \frac{P(s_{t-1} = 1)\Phi(y_{t-1}; y_{t-2}, \hat{\alpha}^{(1)}, \hat{\phi}^{(1)}, \hat{\sigma})}{P(s_{t-1} = 1)\Phi(y_{t-1}; y_{t-2}, \hat{\alpha}^{(1)}, \hat{\phi}^{(1)}, \hat{\sigma}) + P(s_{t-1} = 2)\Phi(y_{t-1}; y_{t-2}, \hat{\alpha}^{(2)}, \hat{\phi}^{(2)}, \hat{\sigma})} \tag{C4}$$

where Φ denotes the probability density function and $P(s_{t-1} = 1)$ represents the prior probability of regime 1 (Hamilton, 1994):

$$P(s_{t-1} = 1) = \frac{1 - p_{22}}{2 - p_{11} - p_{22}}. \tag{C5}$$

Then $P(s_t = 1|y_{t-1})$ and $P(s_t = 2|y_{t-1})$ can be calculated with estimates of the transition matrix:

$$P(s_t = 1|y_{t-1}) = P(s_{t-1} = 1|y_{t-1})p_{11} + P(s_{t-1} = 2|y_{t-1})p_{21}, \tag{C6}$$

$$P(s_t = 2|y_{t-1}) = 1 - P(s_t = 1|y_{t-1}). \tag{C7}$$

As estimating these two models is highly computationally expensive, we only conducted CV for $T = 50, 100, 200$.

Many nonstationary models directly use the time-point variable, t, as a predictor. As the "population" time-series in OOS prediction is much longer than any training samples, the time-point variable will have a much larger range in the former than the latter if we simply assign $t = 1, 2, \ldots, 100000$ to the "population" time-series. This can potentially cause extrapolation problems when using estimates of such prediction models. Therefore, when simulating "population" time-series, we keep the range of the time-point variable always the same as in the training samples. For example, when conducting model selection on samples of 100 time-points, the "population“ is generated by combining 1000 samples, each with 100 predictable time-points (as in Table C5).

Table C5

"Population" time-series when the training sample contains 100 predictable time-points

t	*sample*	y_t	y_{t-1}
0	1	3	NA
1	1	5	3
2	1	4	5
...	1	...	...
100	1	2	4
0	2	5	NA
1	2	3	5
...	...	...	...
0	1000	4	NA
1	1000	3	4
...	1000	...	...
99	1000	4	3
100	1000	5	4

Appendix D

A closer look at the performance of model selection techniques in the simulation study

The workflow of evaluating the performance of one model selection technique in one simulation condition is as follows: first, we rank the nine candidate models according to the model selection results (1 - best, 9 - worst)[3] within each iteration. Then, for each simulation condition, we present three important aspects of each model selection technique's performance in Tables **??** - D7: (1) the average rank of the true model and its standard error (SE), (2) the proportion of iterations where the true model is considered the best model and its SE, and (3) the candidate model with the highest average rank (the "optimal" model based on the model selection technique) and its average rank[4]. The SE of the estimates quantifies the uncertainty in our simulation results due to random sampling of replications. The model selection technique that has the highest average rank for the true model is highlighted in bold.

True model being the random walk model

Table D1 compares the model selection techniques on time-series data in a random walk process. Most ICs show highly similar behaviors to the previous simulation condition: BIC can consistently identify the true model, whereas AIC, AICc, and HQ all tend to overfit in certain sample sizes (i.e., consistently, in large samples, and small samples). As the prediction rule of the random walk model (used in CV and OOS prediction) is always $\hat{y}_t = y_{t-1}$, its prediction error is always identical to the simulated noise term in this simulation condition – as small as it can reasonably get. It is therefore not surprising that blocked CV and OOS predictive accuracy consistently identify the true model in this condition. However, LOOCV prefers the TV-AR(1) model with time-varying intercept and thus overfits.

[3] Models that do not converge get "NA" as its rank.

[4] As CV is not conducted for $T = 1000$, relevant cells are marked as NA in the tables.

Table D1

Performance of model selection techniques when the true model is random walk

T	Criteria	AIC	AICc	HQ	BIC	LOOCV	Blocked CV	OOS prediction
50	rank	3.91 (0.13)	3.19 (0.12)	3.20 (0.11)	1.87 (0.10)	3.82 (0.17)	2.34 (0.15)	**1.00** (0.00)
	proportion	0.01 (0.01)	0.01 (0.01)	0.03 (0.02)	0.45 (0.05)	0.02 (0.01)	0.41 (0.05)	1.00 (0.00)
	optimal	TV-AR(1): α varies (1.24)	TV-AR(1): α varies (1.26)	TV-AR(1): α varies (1.52)	RW (1.87)	TV-AR(1): α varies (1.75)	RW (2.34)	RW (1.00)
100	rank	3.88 (0.14)	3.68 (0.14)	2.73 (0.14)	1.36 (0.07)	3.65 (0.15)	2.16 (0.14)	**1.00** (0.00)
	proportion	0.01 (0.01)	0.01 (0.01)	0.16 (0.04)	0.75 (0.04)	0.03 (0.02)	0.43 (0.05)	1.00 (0.00)
	optimal	TV-AR(1): α varies (1.26)	TV-AR(1): α varies (1.28)	TV-AR(1): α varies (2.15)	RW (1.36)	TV-AR(1): α varies (1.86)	RW (2.16)	RW (1.00)
200	rank	3.81 (0.13)	3.70 (0.13)	2.04 (0.10)	1.04 (0.02)	3.64 (0.13)	1.94 (0.13)	**1.00** (0.00)
	proportion	0.00 (0.00)	0.01 (0.01)	0.36 (0.05)	0.96 (0.02)	0.00 (0.00)	0.55 (0.05)	1.00 (0.00)
	optimal	TV-AR(1): α varies (1.23)	TV-AR(1): α varies (1.25)	RW (2.04)	RW (1.04)	TV-AR(1): α varies (1.89)	RW (1.94)	RW (1.00)
1000	rank	3.95 (0.12)	3.93 (0.12)	1.56 (0.09)	1.01 (0.01)	NA (NA)	NA (NA)	**1.00** (0.00)
	proportion	0.00 (0.00)	0.00 (0.00)	0.63 (0.05)	0.99 (0.01)	NA (NA)	NA (NA)	1.00 (0.00)
	optimal	TV-AR(1): α varies (1.26)	TV-AR(1): α varies (1.25)	RW (1.56)	RW (1.01)	NA (NA)	NA (NA)	RW (1.00)

True model being the SETAR(2,1,1) model

The SETAR(2,1,1) model is one of the most complex candidate models in the simulation study. The sample size required for accurately estimating this model is thus larger, which explains why SETAR(2,1,1) shows worse OOS predictive accuracy than the more parsimonious AR(1) model in small samples (Shmueli, 2010). The two CV techniques perform similarly to OOS prediction. All ICs show overall good performance.

Table D2

Performance of model selection techniques when the true model is SETAR(2,1,1)

Condition	***T***	Criteria	AIC	AICc	HQ	BIC	LOOCV	Blocked CV	OOS prediction
$\alpha^{(1)} = 1, \alpha^{(2)} = 2,$ $\phi^{(1)} = .4,\ \phi^{(2)} = .4,$	50	rank	1.88 (0.10)	3.72 (0.26)	1.29 (0.07)	**1.23** (0.05)	4.83 (0.27)	4.22 (0.24)	4.23 (0.21)
		proportion	0.45 (0.05)	0.28 (0.04)	0.81 (0.04)	0.83 (0.04)	0.21 (0.04)	0.20 (0.04)	0.14 (0.03)
		optimal	SETAR (1.88)	TV-AR(1): α varies (2.83)	SETAR (1.29)	SETAR (1.23)	AR(1) (2.65)	AR(1) (2.15)	AR(1) (1.53)
	100	rank	1.32 (0.07)	1.53 (0.11)	**1.11** (0.04)	1.14 (0.05)	3.22 (0.23)	3.01 (0.19)	2.75 (0.20)
		proportion	0.80 (0.04)	0.75 (0.04)	0.92 (0.03)	0.91 (0.03)	0.44 (0.05)	0.36 (0.05)	0.51 (0.05)
		optimal	SETAR (1.32)	SETAR (1.53)	SETAR (1.11)	SETAR (1.14)	AR(1) (2.84)	AR(1) (2.29)	AR(1) (2.04)
	200	rank	1.19 (0.06)	1.20 (0.07)	**1.11** (0.05)	1.16 (0.06)	1.89 (0.16)	2.02 (0.16)	1.29 (0.10)
		proportion	0.90 (0.03)	0.90 (0.03)	0.94 (0.02)	0.90 (0.03)	0.67 (0.05)	0.63 (0.05)	0.90 (0.03)
		optimal	SETAR (1.19)	SETAR (1.20)	SETAR (1.11)	SETAR (1.16)	SETAR (1.89)	SETAR (2.02)	SETAR (1.29)
	1000	rank	**1.00** (0.00)	**1.00** (0.00)	**1.00** (0.00)	**1.00** (0.00)	NA (NA)	NA (NA)	**1.00** (0.00)
		proportion	1.00 (0.00)	1.00 (0.00)	1.00 (0.00)	1.00 (0.00)	NA (NA)	NA (NA)	1.00 (0.00)
		optimal	SETAR (1.00)	SETAR (1.00)	SETAR (1.00)	SETAR (1.00)	NA (NA)	NA (NA)	SETAR (1.00)
$\alpha^{(1)} = \alpha^{(2)} = 1,$ $\phi^{(1)} = .8,\ \phi^{(2)} = .4,$	50	rank	1.35 (0.08)	2.27 (0.23)	**1.19** (0.06)	1.25 (0.07)	4.16 (0.29)	4.13 (0.29)	2.79 (0.25)
		proportion	0.79 (0.04)	0.64 (0.05)	0.89 (0.03)	0.86 (0.03)	0.34 (0.05)	0.34 (0.05)	0.57 (0.05)
		optimal	SETAR (1.35)	SETAR (2.27)	SETAR (1.19)	SETAR (1.25)	AR(1) (3.14)	AR(1) (3.00)	AR(1) (2.46)
	100	rank	**1.02** (0.01)	**1.02** (0.01)	**1.02** (0.01)	1.05 (0.03)	2.04 (0.22)	2.05 (0.20)	1.23 (0.11)
		proportion	0.98 (0.01)	0.98 (0.01)	0.98 (0.01)	0.96 (0.02)	0.75 (0.04)	0.71 (0.05)	0.93 (0.03)
		optimal	SETAR (1.02)	SETAR (1.02)	SETAR (1.02)	SETAR (1.05)	SETAR (2.04)	SETAR (2.05)	SETAR (1.23)
	200	rank	**1.00** (0.00)	**1.00** (0.00)	**1.00** (0.00)	**1.00** (0.00)	1.14 (0.08)	1.15 (0.09)	**1.00** (0.00)
		proportion	1.00 (0.00)	1.00 (0.00)	1.00 (0.00)	1.00 (0.00)	0.96 (0.02)	0.97 (0.02)	1.00 (0.00)
		optimal	SETAR (1.00)	SETAR (1.00)	SETAR (1.00)	SETAR (1.00)	SETAR (1.14)	SETAR (1.15)	SETAR (1.00)
	1000	rank	**1.00** (0.00)	**1.00** (0.00)	**1.00** (0.00)	**1.00** (0.00)	NA (NA)	NA (NA)	**1.00** (0.00)
		proportion	1.00 (0.00)	1.00 (0.00)	1.00 (0.00)	1.00 (0.00)	NA (NA)	NA (NA)	1.00 (0.00)
		optimal	SETAR (1.00)	SETAR (1.00)	SETAR (1.00)	SETAR (1.00)	NA (NA)	NA (NA)	SETAR (1.00)
$\alpha^{(1)} = 1,\ \alpha^{(2)} = 2,$ $\phi^{(1)} = .8,\ \phi^{(2)} = .4,$	50	rank	2.09 (0.14)	4.58 (0.27)	**1.41** (0.09)	1.53 (0.10)	5.97 (0.22)	5.65 (0.21)	4.96 (0.23)
		proportion	0.53 (0.05)	0.24 (0.04)	0.78 (0.04)	0.69 (0.05)	0.06 (0.02)	0.06 (0.02)	0.11 (0.03)
		optimal	SETAR (2.09)	AR(1) (2.30)	SETAR (1.41)	SETAR (1.53)	AR(1) (2.40)	AR(1) (2.02)	AR(1) (1.80)
	100	rank	1.47 (0.10)	2.05 (0.15)	**1.32** (0.08)	1.59 (0.11)	4.49 (0.24)	4.37 (0.21)	4.52 (0.19)
		proportion	0.75 (0.04)	0.61 (0.05)	0.80 (0.04)	0.64 (0.05)	0.22 (0.04)	0.18 (0.04)	0.13 (0.03)
		optimal	SETAR (1.47)	SETAR (2.05)	SETAR (1.32)	SETAR (1.59)	AR(1) (2.27)	AR(1) (2.07)	AR(1) (1.75)
	200	rank	**1.62** (0.12)	1.81 (0.14)	1.63 (0.12)	1.99 (0.12)	3.64 (0.22)	3.59 (0.21)	3.12 (0.23)
		proportion	0.76 (0.04)	0.71 (0.05)	0.69 (0.05)	0.45 (0.05)	0.34 (0.05)	0.32 (0.05)	0.47 (0.05)
		optimal	SETAR (1.62)	SETAR (1.81)	SETAR (1.63)	AR(1) (1.49)	AR(1) (2.25)	AR(1) (2.17)	AR(1) (2.00)
	1000	rank	**1.00** (0.00)	**1.00** (0.00)	1.04 (0.04)	1.10 (0.05)	NA (NA)	NA (NA)	1.08 (0.06)
		proportion	1.00 (0.00)	1.00 (0.00)	0.99 (0.01)	0.93 (0.03)	NA (NA)	NA (NA)	0.98 (0.01)
		optimal	SETAR (1.00)	SETAR (1.00)	SETAR (1.04)	SETAR (1.10)	NA (NA)	NA (NA)	SETAR (1.08)

Note: For all three simulation conditions, $\sigma^{(1)} = \sigma^{(2)} = 1, \tau = 3$.

True model being the HMM

As indicated by Table D3, the performance of model selection techniques when the true model is the HMM largely depends on the difference between the two regimes. When the difference is large ($\alpha^{(1)} = -2.5, \alpha^{(2)} = 2.5$), all ICs perform well, while the prediction-based techniques show larger variances (i.e., larger SE). When the difference between regimes is small ($\alpha^{(1)} = -.5, \alpha^{(2)} = .5$), the performance of all techniques is

unsatisfactory except for the ICs when $T = 1000$.

Table D3

Performance of model selection techniques when the true model is a hidden Markov model

Condition	Switching	T	Criteria	AIC	AICc	HQ	BIC	LOOCV	Blocked CV	OOS prediction
$\alpha^{(1)} = -2.5, \alpha^{(2)} = 2.5$	at $\frac{T}{2}$	50	rank	1.40 (0.07)	1.26 (0.05)	1.35 (0.06)	**1.24** (0.05)	1.86 (0.19)	1.70 (0.19)	2.36 (0.15)
			proportion	0.71 (0.05)	0.79 (0.04)	0.73 (0.04)	0.80 (0.04)	0.63 (0.05)	0.79 (0.04)	0.01 (0.01)
			optimal	HMM (1.40)	HMM (1.26)	HMM (1.35)	HMM (1.24)	HMM (1.86)	HMM (1.70)	TV-AR(1): α varies (1.03)
		100	rank	1.12 (0.04)	**1.09** (0.03)	1.10 (0.03)	**1.09** (0.03)	1.92 (0.21)	1.56 (0.20)	2.51 (0.18)
			proportion	0.89 (0.03)	0.92 (0.03)	0.90 (0.03)	0.91 (0.03)	0.69 (0.05)	0.91 (0.03)	0.02 (0.01)
			optimal	HMM (1.12)	HMM (1.09)	HMM (1.10)	HMM (1.09)	HMM (1.92)	HMM (1.56)	TV-AR(1): α varies (1.05)
		200	rank	**1.05** (0.02)	**1.05** (0.02)	1.07 (0.03)	1.09 (0.03)	2.03 (0.23)	3.56 (0.33)	2.45 (0.22)
			proportion	0.95 (0.02)	0.95 (0.02)	0.93 (0.03)	0.91 (0.03)	0.67 (0.05)	0.54 (0.05)	0.25 (0.04)
			optimal	HMM (1.05)	HMM (1.05)	HMM (1.07)	HMM (1.09)	HMM (2.03)	SETAR (1.62)	TV-AR(1): α varies (1.34)
		1000	rank	1.02 (0.01)	1.02 (0.01)	**1.00** (0.00)	**1.00** (0.00)	NA (NA)	NA (NA)	3.20 (0.35)
			proportion	0.98 (0.01)	0.98 (0.01)	1.00 (0.00)	1.00 (0.00)	NA (NA)	NA (NA)	0.67 (0.05)
			optimal	HMM (1.02)	HMM (1.02)	HMM (1.00)	HMM (1.00)	NA (NA)	NA (NA)	SETAR (1.86)
	at $\frac{T}{4}, \frac{T}{2}, \frac{3T}{4}$	50	rank	1.16 (0.04)	**1.04** (0.02)	1.12 (0.04)	1.08 (0.03)	1.79 (0.12)	1.10 (0.07)	2.15 (0.04)
			proportion	0.87 (0.03)	0.96 (0.02)	0.89 (0.03)	0.92 (0.03)	0.44 (0.05)	0.97 (0.02)	0.00 (0.00)
			optimal	HMM (1.16)	HMM (1.04)	HMM (1.12)	HMM (1.08)	TV-AR(1): α varies (1.48)	HMM (1.10)	TV-AR(1): α varies (1.00)
		100	rank	**1.02** (0.01)	**1.02** (0.01)	**1.02** (0.01)	1.03 (0.02)	1.26 (0.05)	1.07 (0.03)	2.00 (0.02)
			proportion	0.98 (0.01)	0.98 (0.01)	0.98 (0.01)	0.98 (0.01)	0.77 (0.04)	0.93 (0.03)	0.02 (0.01)
			optimal	HMM (1.02)	HMM (1.02)	HMM (1.02)	HMM (1.03)	HMM (1.26)	HMM (1.07)	TV-AR(1): α varies (1.02)
		200	rank	1.02 (0.01)	1.02 (0.01)	**1.01** (0.01)	**1.01** (0.01)	1.25 (0.05)	2.26 (0.23)	1.06 (0.02)
			proportion	0.98 (0.01)	0.98 (0.01)	0.99 (0.01)	0.99 (0.01)	0.78 (0.04)	0.63 (0.05)	0.94 (0.02)
			optimal	HMM (1.02)	HMM (1.02)	HMM (1.01)	HMM (1.01)	HMM (1.25)	SETAR (1.69)	HMM (1.06)
		1000	rank	1.02 (0.01)	1.02 (0.01)	1.02 (0.01)	**1.01** (0.01)	NA (NA)	NA (NA)	1.11 (0.03)
			proportion	0.98 (0.01)	0.98 (0.01)	0.98 (0.01)	0.99 (0.01)	NA (NA)	NA (NA)	0.89 (0.03)
			optimal	HMM (1.02)	HMM (1.02)	HMM (1.02)	HMM (1.01)	NA (NA)	NA (NA)	HMM (1.11)
$\alpha^{(1)} = -.5, \alpha^{(2)} = .5$	at $\frac{T}{2}$	50	rank	5.24 (0.18)	4.89 (0.17)	5.63 (0.16)	6.06 (0.13)	5.17 (0.23)	**4.62** (0.24)	5.56 (0.19)
			proportion	0.01 (0.01)	0.02 (0.01)	0.01 (0.01)	0.01 (0.01)	0.10 (0.03)	0.16 (0.04)	0.02 (0.01)
			optimal	TV-AR(1): α varies (1.88)	TV-AR(1): α varies (2.00)	SETAR (2.14)	SETAR (2.49)	AR(1) with trend (2.16)	AR(1) with trend (2.25)	AR(1) with trend (1.69)
		100	rank	3.50 (0.13)	**3.46** (0.13)	3.92 (0.16)	4.26 (0.18)	6.52 (0.19)	6.15 (0.23)	6.61 (0.16)
			proportion	0.00 (0.00)	0.00 (0.00)	0.01 (0.01)	0.04 (0.02)	0.05 (0.02)	0.09 (0.03)	0.00 (0.00)
			optimal	TV-AR(1): α varies (1.39)	TV-AR(1): α varies (1.38)	TV-AR(1): α varies (1.87)	AR(1) with trend (1.72)	AR(1) with trend (1.66)	AR(1) with trend (1.55)	TV-AR(1): α varies (1.39)
		200	rank	2.47 (0.07)	2.46 (0.07)	2.28 (0.08)	**2.13** (0.09)	7.44 (0.06)	7.11 (0.14)	7.41 (0.07)
			proportion	0.05 (0.02)	0.06 (0.02)	0.21 (0.04)	0.30 (0.05)	0.00 (0.00)	0.00 (0.00)	0.00 (0.00)
			optimal	TV-AR(1): α varies (1.18)	TV-AR(1): α varies (1.21)	TV-AR(1): α varies (1.75)	AR(1) with trend (1.52)	TV-AR(1): α varies (1.29)	AR(1) with trend (1.39)	TV-AR(1): α varies (1.09)
		1000	rank	1.50 (0.07)	1.50 (0.07)	1.06 (0.02)	**1.00** (0.00)	NA (NA)	NA (NA)	7.48 (0.05)
			proportion	0.59 (0.05)	0.59 (0.05)	0.94 (0.02)	1.00 (0.00)	NA (NA)	NA (NA)	0.00 (0.00)
			optimal	HMM (1.50)	HMM (1.50)	HMM (1.06)	HMM (1.00)	NA (NA)	NA (NA)	TV-AR(1): α varies (1.00)
	at $\frac{T}{4}, \frac{T}{2}, \frac{3T}{4}$	50	rank	5.80 (0.20)	5.60 (0.20)	6.14 (0.17)	6.15 (0.15)	4.10 (0.20)	**3.69** (0.20)	3.86 (0.21)
			proportion	0.02 (0.01)	0.03 (0.02)	0.02 (0.01)	0.02 (0.01)	0.08 (0.03)	0.16 (0.04)	0.17 (0.04)
			optimal	TV-AR(1): α varies (2.05)	TV-AR(1): α varies (2.55)	SETAR (1.72)	SETAR (1.86)	AR(1) (3.39)	AR(1) (2.86)	AR(1) (3.04)
		100	rank	3.76 (0.19)	3.68 (0.19)	4.21 (0.21)	4.38 (0.21)	4.67 (0.22)	**3.25** (0.20)	4.90 (0.21)
			proportion	0.03 (0.02)	0.04 (0.02)	0.08 (0.03)	0.14 (0.03)	0.13 (0.03)	0.23 (0.04)	0.04 (0.02)
			optimal	TV-AR(1): α varies (1.36)	TV-AR(1): α varies (1.42)	TV-AR(1): α varies (2.37)	WN (2.90)	TV-AR(1): α varies (1.90)	HMM (3.25)	TV-AR(1): α varies (1.19)
		200	rank	2.24 (0.05)	2.23 (0.04)	2.00 (0.09)	**1.62** (0.12)	6.34 (0.17)	3.71 (0.24)	6.24 (0.18)
			proportion	0.00 (0.00)	0.00 (0.00)	0.24 (0.04)	0.69 (0.05)	0.02 (0.01)	0.28 (0.04)	0.00 (0.00)
			optimal	TV-AR(1): α varies (1.06)	TV-AR(1): α varies (1.06)	TV-AR(1): α varies (1.43)	HMM (1.62)	TV-AR(1): α varies (1.05)	AR(1) with trend (3.23)	TV-AR(1): α varies (1.00)
		1000	rank	1.31 (0.05)	1.29 (0.05)	1.05 (0.02)	**1.00** (0.00)	NA (NA)	NA (NA)	7.65 (0.05)
			proportion	0.73 (0.04)	0.73 (0.04)	0.95 (0.02)	1.00 (0.00)	NA (NA)	NA (NA)	0.00 (0.00)
			optimal	HMM (1.31)	HMM (1.29)	HMM (1.05)	HMM (1.00)	NA (NA)	NA (NA)	TV-AR(1): α varies (1.00)

True model being the RS-AR(1) model

As shown in Tables D4 and D5, the assessed model selection techniques are not able to consistently select the RS-AR(1) model when it is the true model. When the difference in autocorrelation is small between the two regimes, the prediction-based techniques usually prefer the simpler AR(1) model, whereas the ICs select the SETAR model very often. In conditions with large autocorrelation differences between two regimes, the

TV-AR(1) model with time-varying autocorrelation is often selected when the sample size is large (e.g., $T = 200, 1000$).

Table D4

Performance of model selection techniques when the true model is a regime-switching AR(1) model: $\bar{\phi} = .3$

Condition	Switching	T	Criteria	AIC	AICc	HQ	BIC	LOOCV	Blocked CV	OOS prediction
$\phi^{(1)} = .25, \phi^{(2)} = .35$	at $\frac{T}{2}$	50	rank	6.62 (0.20)	6.81 (0.18)	7.19 (0.17)	7.86 (0.15)	5.01 (0.24)	**4.43** (0.23)	4.56 (0.25)
			proportion	0.05 (0.02)	0.05 (0.02)	0.03 (0.02)	0.02 (0.01)	0.14 (0.03)	0.15 (0.04)	0.22 (0.04)
			optimal	SETAR (2.21)	AR(1) (2.41)	SETAR (1.51)	SETAR (1.86)	AR(1) (2.54)	AR(1) (2.39)	AR(1) (1.90)
		100	rank	6.17 (0.18)	6.41 (0.17)	7.13 (0.14)	7.64 (0.10)	4.78 (0.22)	4.80 (0.21)	**4.25** (0.21)
			proportion	0.04 (0.02)	0.02 (0.01)	0.02 (0.01)	0.00 (0.00)	0.10 (0.03)	0.12 (0.03)	0.19 (0.04)
			optimal	SETAR (2.21)	AR(1) (2.45)	SETAR (1.94)	AR(1) (1.87)	AR(1) (2.06)	AR(1) (2.24)	AR(1) (1.61)
		200	rank	5.50 (0.17)	5.65 (0.16)	6.50 (0.11)	7.23 (0.09)	4.74 (0.20)	**4.55** (0.19)	4.67 (0.18)
			proportion	0.02 (0.01)	0.03 (0.02)	0.00 (0.00)	0.00 (0.00)	0.11 (0.03)	0.12 (0.03)	0.12 (0.03)
			optimal	SETAR (1.78)	SETAR (2.04)	SETAR (1.90)	AR(1) (1.39)	AR(1) (1.86)	AR(1) (1.83)	AR(1) (1.64)
		1000	rank	5.14 (0.15)	5.15 (0.14)	5.84 (0.06)	5.93 (0.04)	NA (NA)	NA (NA)	**4.77** (0.17)
			proportion	0.03 (0.02)	0.03 (0.02)	0.00 (0.00)	0.00 (0.00)	NA (NA)	NA (NA)	0.07 (0.03)
			optimal	SETAR (2.05)	SETAR (2.09)	AR(1) (1.76)	AR(1) (1.16)	NA (NA)	NA (NA)	AR(1) (1.85)
	at $\frac{T}{4}, \frac{T}{2}, \frac{3T}{4}$	50	rank	6.42 (0.22)	6.73 (0.20)	7.14 (0.17)	7.98 (0.09)	5.13 (0.22)	**4.62** (0.24)	4.68 (0.21)
			proportion	0.03 (0.02)	0.05 (0.02)	0.00 (0.00)	0.00 (0.00)	0.08 (0.03)	0.17 (0.04)	0.10 (0.03)
			optimal	SETAR (1.79)	AR(1) (2.60)	SETAR (1.33)	SETAR (1.58)	AR(1) (2.52)	AR(1) (2.57)	AR(1) (1.45)
		100	rank	6.18 (0.18)	6.51 (0.17)	7.30 (0.10)	7.79 (0.05)	4.88 (0.24)	4.65 (0.21)	**4.19** (0.24)
			proportion	0.02 (0.01)	0.02 (0.01)	0.00 (0.00)	0.00 (0.00)	0.17 (0.04)	0.12 (0.03)	0.27 (0.04)
			optimal	SETAR (2.32)	AR(1) (2.42)	SETAR (1.95)	AR(1) (1.81)	AR(1) (2.05)	AR(1) (2.17)	AR(1) (1.69)
		200	rank	5.51 (0.17)	5.56 (0.17)	6.53 (0.12)	7.35 (0.08)	5.15 (0.20)	**4.55** (0.21)	4.91 (0.18)
			proportion	0.02 (0.01)	0.03 (0.02)	0.00 (0.00)	0.00 (0.00)	0.09 (0.03)	0.13 (0.03)	0.09 (0.03)
			optimal	SETAR (2.05)	SETAR (2.39)	AR(1) (1.96)	AR(1) (1.42)	AR(1) (1.94)	AR(1) (1.93)	AR(1) (1.42)
		1000	rank	5.10 (0.16)	5.10 (0.16)	5.79 (0.07)	5.88 (0.04)	NA (NA)	NA (NA)	**4.56** (0.19)
			proportion	0.04 (0.02)	0.04 (0.02)	0.00 (0.00)	0.00 (0.00)	NA (NA)	NA (NA)	0.16 (0.04)
			optimal	SETAR (1.97)	SETAR (2.02)	AR(1) (1.46)	AR(1) (1.08)	NA (NA)	NA (NA)	AR(1) (2.19)
$\phi^{(1)} = .05, \phi^{(2)} = .55$	at $\frac{T}{2}$	50	rank	6.45 (0.19)	6.71 (0.15)	7.31 (0.12)	7.95 (0.07)	5.56 (0.21)	**4.83** (0.21)	**4.83** (0.20)
			proportion	0.01 (0.01)	0.01 (0.01)	0.00 (0.00)	0.00 (0.00)	0.06 (0.02)	0.07 (0.03)	0.05 (0.02)
			optimal	SETAR (2.09)	AR(1) (2.71)	SETAR (1.51)	SETAR (1.69)	AR(1) (2.77)	AR(1) (2.72)	TV-AR(1): ϕ varies (1.75)
		100	rank	5.27 (0.21)	5.41 (0.21)	6.31 (0.17)	7.15 (0.12)	5.09 (0.21)	4.69 (0.19)	**4.60** (0.18)
			proportion	0.05 (0.02)	0.04 (0.02)	0.02 (0.01)	0.00 (0.00)	0.09 (0.03)	0.06 (0.02)	0.01 (0.01)
			optimal	TV-AR(1): ϕ varies (2.17)	TV-AR(1): ϕ varies (2.08)	SETAR (1.94)	AR(1) (2.28)	AR(1) (2.57)	TV-AR(1): ϕ varies (2.48)	TV-AR(1): ϕ varies (1.33)
		200	rank	**3.90** (0.20)	3.93 (0.20)	4.86 (0.20)	6.14 (0.16)	5.19 (0.19)	4.44 (0.18)	4.74 (0.16)
			proportion	0.03 (0.02)	0.02 (0.01)	0.01 (0.01)	0.00 (0.00)	0.05 (0.02)	0.06 (0.02)	0.00 (0.00)
			optimal	TV-AR(1): ϕ varies (1.32)	TV-AR(1): ϕ varies (1.26)	TV-AR(1): ϕ varies (1.45)	TV-AR(1): ϕ varies (1.92)	TV-AR(1): ϕ varies (1.46)	TV-AR(1): ϕ varies (1.84)	TV-AR(1): ϕ varies (1.00)
		1000	rank	1.93 (0.03)	1.93 (0.03)	1.81 (0.04)	**1.61** (0.05)	NA (NA)	NA (NA)	5.99 (0.09)
			proportion	0.07 (0.03)	0.07 (0.03)	0.19 (0.04)	0.39 (0.05)	NA (NA)	NA (NA)	0.00 (0.00)
			optimal	TV-AR(1): ϕ varies (1.07)	TV-AR(1): ϕ varies (1.07)	TV-AR(1): ϕ varies (1.19)	TV-AR(1): ϕ varies (1.39)	NA (NA)	NA (NA)	TV-AR(1): ϕ varies (1.00)
	at $\frac{T}{4}, \frac{T}{2}, \frac{3T}{4}$	50	rank	6.36 (0.20)	6.64 (0.17)	7.14 (0.14)	7.96 (0.10)	5.00 (0.21)	4.91 (0.21)	**4.59** (0.21)
			proportion	0.04 (0.02)	0.03 (0.02)	0.00 (0.00)	0.00 (0.00)	0.07 (0.03)	0.05 (0.02)	0.14 (0.03)
			optimal	SETAR (2.34)	AR(1) (2.72)	SETAR (1.49)	SETAR (1.65)	AR(1) (2.69)	AR(1) (2.46)	AR(1) (1.77)
		100	rank	5.68 (0.18)	5.80 (0.18)	6.69 (0.14)	7.43 (0.09)	4.93 (0.19)	4.35 (0.19)	**4.08** (0.22)
			proportion	0.03 (0.02)	0.02 (0.01)	0.00 (0.00)	0.00 (0.00)	0.06 (0.02)	0.10 (0.03)	0.14 (0.03)
			optimal	SETAR (2.33)	AR(1) (2.99)	SETAR (1.76)	AR(1) (1.85)	AR(1) (2.39)	AR(1) (2.20)	TV-AR(1): ϕ varies (1.89)
		200	rank	**3.93** (0.18)	4.03 (0.18)	5.16 (0.17)	6.15 (0.14)	4.65 (0.18)	4.29 (0.19)	4.49 (0.15)
			proportion	0.02 (0.01)	0.02 (0.01)	0.02 (0.01)	0.01 (0.01)	0.02 (0.01)	0.07 (0.03)	0.00 (0.00)
			optimal	TV-AR(1): ϕ varies (1.51)	TV-AR(1): ϕ varies (1.51)	TV-AR(1): ϕ varies (2.21)	AR(1) (1.59)	TV-AR(1): ϕ varies (1.86)	AR(1) (2.12)	TV-AR(1): ϕ varies (1.32)
		1000	rank	1.98 (0.05)	1.98 (0.05)	1.85 (0.07)	**1.40** (0.09)	NA (NA)	NA (NA)	2.31 (0.09)
			proportion	0.07 (0.03)	0.07 (0.03)	0.23 (0.04)	0.73 (0.04)	NA (NA)	NA (NA)	0.00 (0.00)
			optimal	TV-AR(1): ϕ varies (1.07)	TV-AR(1): ϕ varies (1.07)	TV-AR(1): ϕ varies (1.23)	RS-AR(1) (1.40)	NA (NA)	NA (NA)	TV-AR(1): ϕ varies (1.00)

Table D5

Performance of model selection techniques when the true model is a regime-switching AR(1) model: $\bar{\phi} = .7$

Condition	Switching	T	Criteria	AIC	AICc	HQ	BIC	LOOCV	Blocked CV	OOS prediction
$\phi^{(1)} = .65, \phi^{(2)} = .75$	at $\frac{T}{2}$	50	rank	5.82 (0.15)	6.12 (0.13)	6.59 (0.10)	7.22 (0.08)	4.82 (0.20)	4.11 (0.20)	**3.93** (0.18)
			proportion	0.00 (0.00)	0.00 (0.00)	0.00 (0.00)	0.00 (0.00)	0.07 (0.03)	0.13 (0.03)	0.10 (0.03)
			optimal	TV-AR(1): α varies (2.00)	TV-AR(1): α varies (2.10)	SETAR (1.58)	SETAR (1.66)	AR(1) (2.53)	AR(1) (2.05)	AR(1) (1.43)
		100	rank	5.28 (0.15)	5.48 (0.13)	6.02 (0.11)	6.73 (0.06)	4.34 (0.19)	**4.02** (0.19)	4.11 (0.18)
			proportion	0.04 (0.02)	0.03 (0.02)	0.01 (0.01)	0.00 (0.00)	0.10 (0.03)	0.14 (0.03)	0.13 (0.03)
			optimal	SETAR (2.02)	AR(1) (2.51)	SETAR (1.62)	AR(1) (1.60)	AR(1) (2.09)	AR(1) (1.77)	AR(1) (1.67)
		200	rank	5.25 (0.15)	5.31 (0.15)	5.69 (0.09)	6.07 (0.06)	4.23 (0.19)	3.86 (0.17)	**3.62** (0.18)
			proportion	0.06 (0.02)	0.06 (0.02)	0.00 (0.00)	0.00 (0.00)	0.14 (0.03)	0.13 (0.03)	0.20 (0.04)
			optimal	SETAR (2.17)	SETAR (2.50)	AR(1) (1.90)	AR(1) (1.28)	AR(1) (2.05)	AR(1) (1.91)	AR(1) (1.92)
		1000	rank	5.49 (0.11)	5.49 (0.11)	5.92 (0.04)	5.90 (0.03)	NA (NA)	NA (NA)	**4.55** (0.15)
			proportion	0.01 (0.01)	0.01 (0.01)	0.00 (0.00)	0.00 (0.00)	NA (NA)	NA (NA)	0.02 (0.01)
			optimal	TV-AR(1): ϕ varies (2.08)	TV-AR(1): ϕ varies (2.07)	AR(1) (1.97)	AR(1) (1.32)	NA (NA)	NA (NA)	TV-AR(1): ϕ varies (1.41)
	at $\frac{T}{4}, \frac{T}{2}, \frac{3T}{4}$	50	rank	5.57 (0.17)	6.02 (0.16)	6.60 (0.13)	7.39 (0.08)	4.42 (0.22)	**3.98** (0.18)	4.04 (0.18)
			proportion	0.03 (0.02)	0.03 (0.02)	0.00 (0.00)	0.00 (0.00)	0.14 (0.03)	0.14 (0.03)	0.14 (0.03)
			optimal	TV-AR(1): α varies (1.97)	TV-AR(1): α varies (2.30)	SETAR (1.72)	SETAR (1.83)	AR(1) (2.31)	AR(1) (2.01)	AR(1) (1.49)
		100	rank	5.38 (0.14)	5.50 (0.13)	6.13 (0.10)	6.84 (0.06)	4.44 (0.17)	3.94 (0.16)	**3.78** (0.17)
			proportion	0.02 (0.01)	0.01 (0.01)	0.00 (0.00)	0.00 (0.00)	0.08 (0.03)	0.07 (0.03)	0.14 (0.03)
			optimal	SETAR (2.07)	AR(1) (2.54)	SETAR (1.75)	AR(1) (1.56)	AR(1) (1.99)	AR(1) (1.72)	AR(1) (1.44)
		200	rank	5.22 (0.15)	5.27 (0.15)	5.71 (0.09)	6.10 (0.05)	3.93 (0.20)	4.05 (0.17)	**3.63** (0.19)
			proportion	0.04 (0.02)	0.04 (0.02)	0.00 (0.00)	0.00 (0.00)	0.24 (0.04)	0.13 (0.03)	0.24 (0.04)
			optimal	SETAR (1.81)	SETAR (2.10)	AR(1) (1.85)	AR(1) (1.29)	AR(1) (2.25)	AR(1) (1.83)	AR(1) (1.55)
		1000	rank	5.13 (0.15)	5.14 (0.15)	5.75 (0.08)	5.83 (0.05)	NA (NA)	NA (NA)	**4.69** (0.15)
			proportion	0.02 (0.01)	0.01 (0.01)	0.00 (0.00)	0.00 (0.00)	NA (NA)	NA (NA)	0.02 (0.01)
			optimal	SETAR (2.05)	SETAR (2.05)	AR(1) (1.57)	AR(1) (1.11)	NA (NA)	NA (NA)	AR(1) (2.21)
$\phi^{(1)} = .45, \phi^{(2)} = .95$	at $\frac{T}{2}$	50	rank	6.13 (0.16)	6.19 (0.15)	6.66 (0.12)	7.02 (0.10)	5.02 (0.18)	4.32 (0.18)	**3.98** (0.17)
			proportion	0.03 (0.02)	0.02 (0.01)	0.00 (0.00)	0.00 (0.00)	0.05 (0.02)	0.09 (0.03)	0.04 (0.02)
			optimal	TV-AR(1): α varies (1.66)	TV-AR(1): α varies (1.77)	SETAR (1.90)	SETAR (1.88)	TV-AR(1): α varies (2.73)	AR(1) (2.42)	TV-AR(1): ϕ varies (2.10)
		100	rank	5.94 (0.13)	6.07 (0.13)	6.45 (0.10)	6.87 (0.06)	4.86 (0.15)	3.88 (0.17)	**3.72** (0.18)
			proportion	0.00 (0.00)	0.00 (0.00)	0.00 (0.00)	0.00 (0.00)	0.03 (0.02)	0.09 (0.03)	0.07 (0.03)
			optimal	TV-AR(1): α varies (1.84)	TV-AR(1): α varies (1.95)	SETAR (2.19)	SETAR (2.07)	TV-AR(1): ϕ varies (2.47)	AR(1) (2.90)	TV-AR(1): ϕ varies (1.83)
		200	rank	5.33 (0.17)	5.39 (0.17)	5.85 (0.16)	6.31 (0.12)	4.73 (0.13)	3.87 (0.18)	**3.46** (0.14)
			proportion	0.00 (0.00)	0.00 (0.00)	0.00 (0.00)	0.00 (0.00)	0.02 (0.01)	0.07 (0.03)	0.02 (0.01)
			optimal	TV-AR(1): ϕ varies (1.40)	TV-AR(1): ϕ varies (1.37)	TV-AR(1): ϕ varies (1.42)	TV-AR(1): ϕ varies (1.83)	TV-AR(1): ϕ varies (1.41)	TV-AR(1): ϕ varies (2.05)	TV-AR(1): ϕ varies (1.47)
		1000	rank	5.12 (0.17)	5.11 (0.18)	5.12 (0.19)	5.14 (0.20)	NA (NA)	NA (NA)	**4.27** (0.13)
			proportion	0.05 (0.02)	0.06 (0.02)	0.12 (0.03)	0.16 (0.04)	NA (NA)	NA (NA)	0.00 (0.00)
			optimal	TV-AR(1): ϕ varies (1.05)	TV-AR(1): ϕ varies (1.06)	TV-AR(1): ϕ varies (1.12)	TV-AR(1): ϕ varies (1.16)	NA (NA)	NA (NA)	TV-AR(1): ϕ varies (1.00)
	at $\frac{T}{4}, \frac{T}{2}, \frac{3T}{4}$	50	rank	5.55 (0.17)	5.72 (0.15)	6.28 (0.13)	6.98 (0.11)	4.84 (0.19)	4.35 (0.16)	**4.01** (0.19)
			proportion	0.01 (0.01)	0.01 (0.01)	0.01 (0.01)	0.00 (0.00)	0.06 (0.02)	0.06 (0.02)	0.12 (0.03)
			optimal	TV-AR(1): α varies (2.13)	TV-AR(1): α varies (2.10)	SETAR (1.75)	SETAR (1.86)	AR(1) (2.41)	AR(1) (1.96)	AR(1) (2.07)
		100	rank	5.78 (0.13)	5.92 (0.13)	6.46 (0.10)	6.81 (0.05)	4.34 (0.17)	3.94 (0.18)	**3.84** (0.18)
			proportion	0.00 (0.00)	0.01 (0.01)	0.00 (0.00)	0.00 (0.00)	0.04 (0.02)	0.14 (0.03)	0.11 (0.03)
			optimal	TV-AR(1): α varies (1.91)	TV-AR(1): α varies (1.96)	SETAR (1.81)	SETAR (1.79)	TV-AR(1): α varies (2.95)	AR(1) (2.26)	AR(1) (2.36)
		200	rank	5.10 (0.17)	5.13 (0.17)	5.68 (0.17)	6.10 (0.14)	4.44 (0.15)	3.71 (0.16)	**3.15** (0.15)
			proportion	0.02 (0.01)	0.02 (0.01)	0.01 (0.01)	0.00 (0.00)	0.02 (0.01)	0.10 (0.03)	0.08 (0.03)
			optimal	TV-AR(1): ϕ varies (1.58)	TV-AR(1): ϕ varies (1.59)	SETAR (1.98)	SETAR (1.77)	TV-AR(1): ϕ varies (1.86)	AR(1) (2.53)	TV-AR(1): ϕ varies (1.89)
		1000	rank	4.48 (0.21)	4.48 (0.21)	4.43 (0.23)	4.27 (0.24)	NA (NA)	NA (NA)	**4.10** (0.15)
			proportion	0.07 (0.03)	0.07 (0.03)	0.21 (0.04)	0.33 (0.05)	NA (NA)	NA (NA)	0.00 (0.00)
			optimal	TV-AR(1): ϕ varies (1.07)	TV-AR(1): ϕ varies (1.07)	TV-AR(1): ϕ varies (1.21)	TV-AR(1): ϕ varies (1.35)	NA (NA)	NA (NA)	TV-AR(1): ϕ varies (1.00)

True model being the TV-AR(1) model with time-varying intercept

As displayed in Table D6, the ICs perform generally well when the true model is the TV-AR(1) model with a time-varying intercept. However, BIC occasionally selects the wrong model in conditions with small sample sizes and small differences in the intercepts over time. Between the two CV techniques, LOOCV consistently shows better performance than 10-block CV. This is partly an over-extrapolation problem: CV entails predicting observations with a different range on the time-point variable from the training observations. Since TV-AR(1) models have smooth functions of time as a covariate, predicting for only one such observation (LOOCV) naturally suffers less from

over-extrapolation than predicting for multiple consecutive observations (10-block CV). The performance of OOS prediction improves as the sample size increases. This suggests that accurately approximating such a sine function requires a large sample size, which is consistent with findings of Bringmann et al. (2017).

Table D6

Performance of model selection techniques when the true model is time-varying AR(1) model: α varies

Condition	T	Criteria	AIC	AICc	HQ	BIC	LOOCV	Blocked CV	OOS prediction
$\alpha_t = .5sin\frac{2\pi t}{T}, \phi = .3$	50	rank	**2.05** (0.13)	2.18 (0.12)	2.90 (0.15)	4.02 (0.19)	3.20 (0.20)	5.39 (0.29)	3.25 (0.28)
		proportion	0.48 (0.05)	0.35 (0.05)	0.17 (0.04)	0.09 (0.03)	0.25 (0.04)	0.09 (0.03)	0.43 (0.05)
		optimal	TV-AR(1): α varies (2.05)	TV-AR(1): α varies (2.18)	SETAR (1.92)	SETAR (1.99)	AR(1) with trend (2.31)	AR(1) (2.37)	AR(1) with trend (1.85)
	100	rank	1.59 (0.11)	**1.56** (0.10)	2.21 (0.12)	3.62 (0.15)	1.91 (0.11)	3.89 (0.23)	1.99 (0.16)
		proportion	0.64 (0.05)	0.65 (0.05)	0.32 (0.05)	0.04 (0.02)	0.46 (0.05)	0.18 (0.04)	0.56 (0.05)
		optimal	TV-AR(1): α varies (1.59)	TV-AR(1): α varies (1.56)	TV-AR(1): α varies (2.21)	AR(1) with trend (2.03)	TV-AR(1): α varies (1.91)	AR(1) with trend (2.02)	AR(1) with trend (1.67)
	200	rank	**1.10** (0.04)	1.12 (0.04)	1.33 (0.07)	2.42 (0.12)	1.27 (0.06)	2.07 (0.18)	1.23 (0.08)
		proportion	0.92 (0.03)	0.90 (0.03)	0.73 (0.04)	0.19 (0.04)	0.79 (0.04)	0.57 (0.05)	0.87 (0.03)
		optimal	TV-AR(1): α varies (1.10)	TV-AR(1): α varies (1.12)	TV-AR(1): α varies (1.33)	AR(1) with trend (1.48)	TV-AR(1): α varies (1.27)	AR(1) with trend (2.04)	TV-AR(1): α varies (1.23)
	1000	rank	**1.00** (0.00)	**1.00** (0.00)	**1.00** (0.00)	1.03 (0.02)	NA (NA)	NA (NA)	**1.00** (0.00)
		proportion	1.00 (0.00)	1.00 (0.00)	1.00 (0.00)	0.97 (0.02)	NA (NA)	NA (NA)	1.00 (0.00)
		optimal	TV-AR(1): α varies (1.00)	TV-AR(1): α varies (1.00)	TV-AR(1): α varies (1.00)	TV-AR(1): α varies (1.03)	NA (NA)	NA (NA)	TV-AR(1): α varies (1.00)
$\alpha_t = 2.5sin\frac{2\pi t}{T}, \phi = .3$	50	rank	1.01 (0.01)	**1.00** (0.00)	1.01 (0.01)	1.14 (0.04)	**1.00** (0.00)	3.58 (0.28)	1.93 (0.20)
		proportion	0.99 (0.01)	1.00 (0.00)	0.99 (0.01)	0.90 (0.03)	1.00 (0.00)	0.49 (0.05)	0.80 (0.04)
		optimal	TV-AR(1): α varies (1.01)	TV-AR(1): α varies (1.00)	TV-AR(1): α varies (1.01)	TV-AR(1): α varies (1.14)	TV-AR(1): α varies (1.00)	RW (2.55)	AR(1) with trend (1.89)
	100	rank	**1.00** (0.00)	**1.00** (0.00)	**1.00** (0.00)	**1.00** (0.00)	**1.00** (0.00)	2.22 (0.22)	**1.00** (0.00)
		proportion	1.00 (0.00)	1.00 (0.00)	1.00 (0.00)	1.00 (0.00)	1.00 (0.00)	0.72 (0.04)	1.00 (0.00)
		optimal	TV-AR(1): α varies (1.00)	TV-AR(1): α varies (1.00)	TV-AR(1): α varies (1.00)	TV-AR(1): α varies (1.00)	TV-AR(1): α varies (1.00)	TV-AR(1): α varies (2.22)	TV-AR(1): α varies (1.00)
	200	rank	**1.00** (0.00)	**1.00** (0.00)	**1.00** (0.00)	**1.00** (0.00)	**1.00** (0.00)	1.13 (0.08)	**1.00** (0.00)
		proportion	1.00 (0.00)	1.00 (0.00)	1.00 (0.00)	1.00 (0.00)	1.00 (0.00)	0.96 (0.02)	1.00 (0.00)
		optimal	TV-AR(1): α varies (1.00)	TV-AR(1): α varies (1.00)	TV-AR(1): α varies (1.00)	TV-AR(1): α varies (1.00)	TV-AR(1): α varies (1.00)	TV-AR(1): α varies (1.13)	TV-AR(1): α varies (1.00)
	1000	rank	**1.00** (0.00)	**1.00** (0.00)	**1.00** (0.00)	**1.00** (0.00)	NA (NA)	NA (NA)	**1.00** (0.00)
		proportion	1.00 (0.00)	1.00 (0.00)	1.00 (0.00)	1.00 (0.00)	NA (NA)	NA (NA)	1.00 (0.00)
		optimal	TV-AR(1): α varies (1.00)	TV-AR(1): α varies (1.00)	TV-AR(1): α varies (1.00)	TV-AR(1): α varies (1.00)	NA (NA)	NA (NA)	TV-AR(1): α varies (1.00)
$\alpha_t = .5sin\frac{2\pi t}{T}, \phi = .7$	50	rank	**1.25** (0.06)	1.37 (0.07)	1.83 (0.12)	4.00 (0.18)	2.14 (0.18)	6.03 (0.24)	6.61 (0.18)
		proportion	0.83 (0.04)	0.73 (0.04)	0.53 (0.05)	0.07 (0.03)	0.61 (0.05)	0.09 (0.03)	0.04 (0.02)
		optimal	TV-AR(1): α varies (1.25)	TV-AR(1): α varies (1.37)	TV-AR(1): α varies (1.83)	SETAR (2.13)	TV-AR(1): α varies (2.14)	AR(1) (2.29)	AR(1) with trend (2.06)
	100	rank	**1.30** (0.07)	1.37 (0.08)	2.09 (0.13)	4.55 (0.16)	2.24 (0.19)	5.35 (0.23)	3.90 (0.26)
		proportion	0.79 (0.04)	0.77 (0.04)	0.42 (0.05)	0.06 (0.02)	0.54 (0.05)	0.11 (0.03)	0.30 (0.05)
		optimal	TV-AR(1): α varies (1.30)	TV-AR(1): α varies (1.37)	TV-AR(1): α varies (2.09)	AR(1) (2.47)	TV-AR(1): α varies (2.24)	AR(1) (2.12)	AR(1) with trend (1.69)
	200	rank	**1.09** (0.04)	1.10 (0.04)	1.52 (0.09)	3.28 (0.17)	1.40 (0.10)	3.84 (0.25)	2.33 (0.20)
		proportion	0.93 (0.03)	0.92 (0.03)	0.67 (0.05)	0.13 (0.03)	0.82 (0.04)	0.29 (0.05)	0.60 (0.05)
		optimal	TV-AR(1): α varies (1.09)	TV-AR(1): α varies (1.10)	TV-AR(1): α varies (1.52)	AR(1) with trend (1.85)	TV-AR(1): α varies (1.40)	AR(1) with trend (2.20)	AR(1) with trend (1.74)
	1000	rank	**1.00** (0.00)	**1.00** (0.00)	**1.00** (0.00)	1.05 (0.02)	NA (NA)	NA (NA)	**1.00** (0.00)
		proportion	1.00 (0.00)	1.00 (0.00)	1.00 (0.00)	0.95 (0.02)	NA (NA)	NA (NA)	1.00 (0.00)
		optimal	TV-AR(1): α varies (1.00)	TV-AR(1): α varies (1.00)	TV-AR(1): α varies (1.00)	TV-AR(1): α varies (1.05)	NA (NA)	NA (NA)	TV-AR(1): α varies (1.00)
$\alpha_t = 2.5sin\frac{2\pi t}{T}, \phi = .7$	50	rank	**1.00** (0.00)	**1.00** (0.00)	**1.00** (0.00)	1.08 (0.03)	**1.00** (0.00)	4.36 (0.26)	5.31 (0.24)
		proportion	1.00 (0.00)	1.00 (0.00)	1.00 (0.00)	0.93 (0.03)	1.00 (0.00)	0.27 (0.04)	0.14 (0.03)
		optimal	TV-AR(1): α varies (1.00)	TV-AR(1): α varies (1.00)	TV-AR(1): α varies (1.00)	TV-AR(1): α varies (1.08)	TV-AR(1): α varies (1.00)	RW (1.50)	TV-AR(1): ϕ varies (1.21)
	100	rank	**1.00** (0.00)	**1.00** (0.00)	1.01 (0.01)	1.63 (0.08)	**1.00** (0.00)	4.43 (0.26)	3.10 (0.26)
		proportion	1.00 (0.00)	1.00 (0.00)	0.99 (0.01)	0.54 (0.05)	1.00 (0.00)	0.29 (0.05)	0.54 (0.05)
		optimal	TV-AR(1): α varies (1.00)	TV-AR(1): α varies (1.00)	TV-AR(1): α varies (1.01)	RW (1.61)	TV-AR(1): α varies (1.00)	RW (1.43)	TV-AR(1): ϕ varies (1.60)
	200	rank	**1.00** (0.00)	**1.00** (0.00)	**1.00** (0.00)	1.34 (0.05)	**1.00** (0.00)	3.55 (0.27)	1.21 (0.10)
		proportion	1.00 (0.00)	1.00 (0.00)	1.00 (0.00)	0.69 (0.05)	1.00 (0.00)	0.51 (0.05)	0.95 (0.02)
		optimal	TV-AR(1): α varies (1.00)	TV-AR(1): α varies (1.00)	TV-AR(1): α varies (1.00)	TV-AR(1): α varies (1.34)	TV-AR(1): α varies (1.00)	RW (1.75)	TV-AR(1): α varies (1.21)
	1000	rank	**1.00** (0.00)	**1.00** (0.00)	**1.00** (0.00)	**1.00** (0.00)	NA (NA)	NA (NA)	**1.00** (0.00)
		proportion	1.00 (0.00)	1.00 (0.00)	1.00 (0.00)	1.00 (0.00)	NA (NA)	NA (NA)	1.00 (0.00)
		optimal	TV-AR(1): α varies (1.00)	TV-AR(1): α varies (1.00)	TV-AR(1): α varies (1.00)	TV-AR(1): α varies (1.00)	NA (NA)	NA (NA)	TV-AR(1): α varies (1.00)

True model being the TV-AR(1) model with time-varying autocorrelation

Correct model selection for time-series data generated by a TV-AR(1) model with time-varying autocorrelation is considerably more challenging as shown in Table D7. Even in large samples, small changes in autocorrelation are not correctly identified: BIC, HQ, and other prediction-focused techniques ignore it by selecting the AR(1) model, while AIC and AICc misinterpreted it as a threshold process. Large changes in autocorrelation can often be correctly detected by all techniques, although not consistently.

Table D7

Performance of model selection techniques when the true model is time-varying AR(1) model: ϕ varies

Condition	***T***	Criteria	AIC	AICc	HQ	BIC	LOOCV	Blocked CV	OOS prediction
$\phi_t = .05sin\frac{2\pi t}{T} + .3$	50	rank	4.41 (0.18)	4.04 (0.17)	4.79 (0.15)	4.95 (0.14)	4.33 (0.21)	4.16 (0.23)	**3.80** (0.19)
		proportion	0.07 (0.03)	0.09 (0.03)	0.01 (0.01)	0.00 (0.00)	0.10 (0.03)	0.19 (0.04)	0.08 (0.03)
		optimal	SETAR (2.11)	AR(1) (2.45)	SETAR (1.29)	SETAR (1.54)	AR(1) (2.58)	AR(1) (2.42)	AR(1) (1.55)
	100	rank	4.24 (0.18)	3.99 (0.17)	4.41 (0.15)	4.71 (0.14)	3.96 (0.20)	3.91 (0.21)	**3.36** (0.16)
		proportion	0.09 (0.03)	0.08 (0.03)	0.01 (0.01)	0.01 (0.01)	0.13 (0.03)	0.13 (0.03)	0.12 (0.03)
		optimal	SETAR (2.03)	AR(1) (2.52)	SETAR (1.76)	AR(1) (1.92)	AR(1) (2.24)	AR(1) (2.17)	AR(1) (1.43)
	200	rank	3.52 (0.16)	3.43 (0.16)	3.56 (0.14)	3.92 (0.14)	**3.17** (0.16)	3.20 (0.18)	3.46 (0.17)
		proportion	0.16 (0.04)	0.19 (0.04)	0.11 (0.03)	0.03 (0.02)	0.18 (0.04)	0.18 (0.04)	0.13 (0.03)
		optimal	SETAR (2.05)	SETAR (2.39)	AR(1) (1.86)	AR(1) (1.29)	AR(1) (1.92)	AR(1) (1.96)	AR(1) (1.38)
	1000	rank	3.18 (0.15)	3.18 (0.15)	3.57 (0.12)	3.60 (0.13)	NA (NA)	NA (NA)	**2.92** (0.17)
		proportion	0.15 (0.04)	0.15 (0.04)	0.03 (0.02)	0.01 (0.01)	NA (NA)	NA (NA)	0.31 (0.05)
		optimal	SETAR (1.94)	SETAR (1.99)	AR(1) (1.39)	AR(1) (1.11)	NA (NA)	NA (NA)	AR(1) (1.77)
$\phi_t = .25sin\frac{2\pi t}{T} + .3$	50	rank	4.01 (0.19)	3.64 (0.19)	4.28 (0.18)	4.42 (0.17)	4.25 (0.22)	3.94 (0.24)	**2.43** (0.16)
		proportion	0.09 (0.03)	0.19 (0.04)	0.05 (0.02)	0.04 (0.02)	0.15 (0.04)	0.23 (0.04)	0.39 (0.05)
		optimal	SETAR (2.29)	AR(1) (2.97)	SETAR (1.58)	SETAR (1.68)	AR(1) (2.98)	AR(1) (2.83)	AR(1) (2.10)
	100	rank	2.47 (0.16)	2.35 (0.15)	2.92 (0.16)	3.62 (0.17)	2.49 (0.18)	2.46 (0.18)	**1.49** (0.12)
		proportion	0.39 (0.05)	0.41 (0.05)	0.25 (0.04)	0.14 (0.03)	0.45 (0.05)	0.41 (0.05)	0.80 (0.04)
		optimal	SETAR (2.16)	TV-AR(1): ϕ varies (2.35)	SETAR (1.84)	AR(1) (1.98)	AR(1) (2.48)	TV-AR(1): ϕ varies (2.46)	TV-AR(1): ϕ varies (1.49)
	200	rank	2.00 (0.13)	1.95 (0.13)	2.45 (0.16)	3.21 (0.19)	1.88 (0.14)	2.53 (0.19)	**1.16** (0.07)
		proportion	0.52 (0.05)	0.54 (0.05)	0.44 (0.05)	0.25 (0.04)	0.60 (0.05)	0.49 (0.05)	0.94 (0.02)
		optimal	TV-AR(1): ϕ varies (2.00)	TV-AR(1): ϕ varies (1.95)	SETAR (2.26)	AR(1) (1.67)	TV-AR(1): ϕ varies (1.88)	AR(1) (2.52)	TV-AR(1): ϕ varies (1.16)
	1000	rank	**1.00** (0.00)	**1.00** (0.00)	1.01 (0.01)	1.46 (0.11)	NA (NA)	NA (NA)	4.32 (0.16)
		proportion	1.00 (0.00)	1.00 (0.00)	0.99 (0.01)	0.80 (0.04)	NA (NA)	NA (NA)	0.11 (0.03)
		optimal	TV-AR(1): ϕ varies (1.00)	TV-AR(1): ϕ varies (1.00)	TV-AR(1): ϕ varies (1.01)	TV-AR(1): ϕ varies (1.46)	NA (NA)	NA (NA)	AR(1) (1.50)
$\phi_t = .05sin\frac{2\pi t}{T} + .7$	50	rank	4.36 (0.14)	3.80 (0.14)	4.57 (0.14)	4.90 (0.14)	4.15 (0.18)	3.46 (0.19)	**3.10** (0.16)
		proportion	0.02 (0.01)	0.07 (0.03)	0.02 (0.01)	0.00 (0.00)	0.08 (0.03)	0.14 (0.03)	0.18 (0.04)
		optimal	SETAR (2.07)	TV-AR(1): α varies (2.38)	SETAR (1.58)	SETAR (1.63)	AR(1) (2.34)	AR(1) (2.01)	AR(1) (1.58)
	100	rank	3.77 (0.14)	3.57 (0.14)	3.85 (0.12)	4.08 (0.12)	3.68 (0.14)	3.44 (0.17)	**3.15** (0.17)
		proportion	0.07 (0.03)	0.08 (0.03)	0.03 (0.02)	0.02 (0.01)	0.07 (0.03)	0.08 (0.03)	0.22 (0.04)
		optimal	SETAR (2.11)	AR(1) (2.34)	SETAR (1.71)	AR(1) (1.55)	AR(1) (1.88)	AR(1) (1.63)	AR(1) (1.54)
	200	rank	3.35 (0.15)	3.28 (0.15)	3.44 (0.14)	3.60 (0.13)	**3.11** (0.14)	3.26 (0.15)	3.15 (0.15)
		proportion	0.14 (0.03)	0.16 (0.04)	0.09 (0.03)	0.02 (0.01)	0.12 (0.03)	0.13 (0.03)	0.17 (0.04)
		optimal	SETAR (2.31)	AR(1) (2.54)	AR(1) (1.79)	AR(1) (1.29)	AR(1) (2.07)	AR(1) (1.82)	AR(1) (1.54)
	1000	rank	2.90 (0.16)	2.88 (0.16)	3.26 (0.15)	3.66 (0.14)	NA (NA)	NA (NA)	**2.59** (0.17)
		proportion	0.24 (0.04)	0.24 (0.04)	0.18 (0.04)	0.05 (0.02)	NA (NA)	NA (NA)	0.49 (0.05)
		optimal	SETAR (1.80)	SETAR (1.83)	AR(1) (1.60)	AR(1) (1.12)	NA (NA)	NA (NA)	AR(1) (1.89)
$\phi_t = .25sin\frac{2\pi t}{T} + .7$	50	rank	3.42 (0.14)	3.03 (0.14)	3.66 (0.15)	4.14 (0.15)	3.25 (0.17)	3.24 (0.20)	**2.24** (0.17)
		proportion	0.05 (0.02)	0.15 (0.04)	0.04 (0.02)	0.04 (0.02)	0.16 (0.04)	0.24 (0.04)	0.54 (0.05)
		optimal	TV-AR(1): α varies (2.02)	TV-AR(1): α varies (2.16)	SETAR (1.74)	SETAR (1.79)	AR(1) (2.95)	AR(1) (2.29)	TV-AR(1): ϕ varies (2.24)
	100	rank	2.48 (0.12)	2.36 (0.12)	2.66 (0.14)	2.97 (0.16)	2.54 (0.15)	2.73 (0.19)	**1.53** (0.11)
		proportion	0.23 (0.04)	0.26 (0.04)	0.23 (0.04)	0.22 (0.04)	0.31 (0.05)	0.41 (0.05)	0.75 (0.04)
		optimal	TV-AR(1): α varies (2.37)	TV-AR(1): ϕ varies (2.36)	SETAR (2.06)	SETAR (2.30)	TV-AR(1): ϕ varies (2.54)	AR(1) (2.43)	TV-AR(1): ϕ varies (1.53)
	200	rank	1.62 (0.09)	1.55 (0.09)	1.70 (0.11)	2.46 (0.15)	1.75 (0.12)	1.96 (0.17)	**1.03** (0.02)
		proportion	0.58 (0.05)	0.63 (0.05)	0.58 (0.05)	0.33 (0.05)	0.57 (0.05)	0.67 (0.05)	0.98 (0.01)
		optimal	TV-AR(1): ϕ varies (1.62)	TV-AR(1): ϕ varies (1.55)	TV-AR(1): ϕ varies (1.70)	SETAR (2.14)	TV-AR(1): ϕ varies (1.75)	TV-AR(1): ϕ varies (1.96)	TV-AR(1): ϕ varies (1.03)
	1000	rank	**1.00** (0.00)	**1.00** (0.00)	**1.00** (0.00)	1.02 (0.01)	NA (NA)	NA (NA)	**1.00** (0.00)
		proportion	1.00 (0.00)	1.00 (0.00)	1.00 (0.00)	0.98 (0.01)	NA (NA)	NA (NA)	1.00 (0.00)
		optimal	TV-AR(1): ϕ varies (1.00)	TV-AR(1): ϕ varies (1.00)	TV-AR(1): ϕ varies (1.00)	TV-AR(1): ϕ varies (1.02)	NA (NA)	NA (NA)	TV-AR(1): ϕ varies (1.00)

Appendix E

*

References

Berkhout, S. W., Schuurman, N. K., & Hamaker, E. L. (2023). A tool to simulate and visualize dyadic interaction dynamics. *Psychological Methods*. https://doi.org/10.1037/met0000575

Bringmann, L. F., Hamaker, E. L., Vigo, D. E., Aubert, A., Borsboom, D., & Tuerlinckx, F. (2017). Changing dynamics: Time-varying autoregressive models using generalized additive modeling. *Psychological Methods*, *22*(3), 409–425. https://doi.org/10.1037/met0000085

Cabrieto, J., Adolf, J., Tuerlinckx, F., Kuppens, P., & Ceulemans, E. (2018). Detecting long-lived autodependency changes in a multivariate system via change point detection and regime switching models. *Scientific Reports*, *8*(1), 15637. https://doi.org/10.1038/s41598-018-33819-8

Dahlhaus, R. (1997). Fitting time series models to nonstationary processes. *The Annals of Statistics*, *25*(1). https://doi.org/10.1214/aos/1034276620

Hamaker, E. L., & Grasman, R. P. P. P. (2012). Regime switching state-space models applied to psychological processes: Handling missing data and making inferences. *Psychometrika*, *77*(2), 400–422. https://doi.org/10.1007/s11336-012-9254-8

Hamaker, E. L., Zhang, Z., & Van Der Maas, H. L. J. (2009). Using threshold autoregressive models to study dyadic interactions. *Psychometrika*, *74*(4), 727. https://doi.org/10.1007/s11336-009-9113-4

Hamilton, J. D. (1994). *Time series analysis*. Princeton University Press. https://doi.org/10.2307/j.ctv14jx6sm

Hastie, T., & Tibshirani, R. (1999). *Generalized additive models* (First CRC reprint). Chapman & Hall/CRC.

Konishi, S., & Kitagawa, G. (2008). *Information criteria and statistical modeling*. Springer.

Ou, L., Hunter, M., D., & Chow, S.-M. (2019). Whats for dynr: A package for linear and nonlinear dynamic modeling in r. *The R Journal*, *11*(1), 91. https://doi.org/10.32614/RJ-2019-012

R Core Team. (2021). *R: A language and environment for statistical computing* (manual). R Foundation for Statistical Computing. Vienna, Austria. https://www.R-project.org/

Shmueli, G. (2010). To explain or to predict? *Statistical Science*, *25*(3). https://doi.org/10.1214/10-STS330

Soch, J., Proofs, T. B. o. S., Maja, Monticone, P., Faulkenberry, T. J., Kipnis, A., Petrykowski, K., Allefeld, C., Atze, H., Knapp, A., McInerney, C. D., Lo4ding00, & amvosk. (2024, January 12). *StatProofBook/StatProofBook.github.io: StatProofBook 2023* (Version 2023). Zenodo. https://doi.org/10.5281/zenodo.10495684

Wong, C. S., & Li, W. K. (1998). A note on the corrected akaike information criterion for threshold autoregressive models. *Journal of Time Series Analysis*, *19*(1), 113–124. https://doi.org/10.1111/1467-9892.00080

Wood, S. N., Pya, N., & Säfken, B. (2016). Smoothing parameter and model selection for general smooth models. *Journal of the American Statistical Association*, *111*(516), 1548–1563. https://doi.org/10.1080/01621459.2016.1180986

Zucchini, W., & MacDonald, I. L. (2009, April 24). *Hidden markov models for time series: An introduction using r*. Chapman; Hall/CRC. https://doi.org/10.1201/9781420010893
